# Global burden of hypoglycaemia-related mortality in 109 countries, from 2000 to 2014: an analysis of death certificates

**DOI:** 10.1007/s00125-018-4626-y

**Published:** 2018-05-01

**Authors:** Francesco Zaccardi, Nafeesa N. Dhalwani, David R. Webb, Melanie J. Davies, Kamlesh Khunti

**Affiliations:** Diabetes Research Centre, University of Leicester, Leicester General Hospital, Gwendolen Rd, Leicester, LE5 4PW UK

**Keywords:** Death certificate, Epidemiology, Global, Hypoglycaemia, Mortality, Trends

## Abstract

**Aims/hypothesis:**

In the context of increasing prevalence of diabetes in elderly people with multimorbidity, intensive glucose control may increase the risk of severe hypoglycaemia, potentially leading to death. While rising trends of severe hypoglycaemia rates have been reported in some European, North American and Asian countries, the global burden of hypoglycaemia-related mortality is unknown. We aimed to investigate global differences and trends of hypoglycaemia-related mortality.

**Methods:**

We used the WHO mortality database to extract information on death certificates reporting hypoglycaemia or diabetes as the underlying cause of death, and the United Nations demographic database to obtain data on mid-year population estimates from 2000 to 2014. We calculated crude and age-standardised proportions (defined as number of hypoglycaemia-related deaths divided by total number of deaths from diabetes [i.e. the sum of hypoglycaemia- and diabetes-related deaths]) and rates (hypoglycaemia-related deaths divided by mid-year population) of hypoglycaemia-related mortality and compared estimates across countries and over time.

**Results:**

Data for proportions were extracted from 109 countries (31 had data from all years analysed [2000–2014] available). Combining all countries, the age-standardised proportion of hypoglycaemia-related deaths was 4.49 (95% CI 4.44, 4.55) per 1000 total diabetes deaths. Compared with the overall mean, most Central American, South American and (mainly) Caribbean countries reported higher proportions (five more age-standardised hypoglycaemia-related deaths per 1000 total diabetes deaths in Chile, six in Uruguay, 11 in Belize and 22 in Aruba), as well as Japan (11 more age-standardised hypoglycaemia-related deaths per 1000 total diabetes deaths). In comparison, lower proportions were noted in most European countries, the USA, Canada, New Zealand and Australia. For countries with data available for all years analysed, trend analysis showed a 60% increase in hypoglycaemia-related deaths until 2010 and stable trends onwards. Rising trends were most evident for Argentina, Brazil, Chile, the USA and Japan. Data for rates were available for 105 countries (30 had data for all years analysed [2000–2014] available). Combining all countries, the age-standardised hypoglycaemia-related death rate was 0.79 (95% CI 0.77, 0.80) per 1 million person-years. Most Central American, South American and Caribbean countries similarly reported higher rates of hypoglycaemia-related death, whilst virtually all European countries, the USA, Canada, Japan, New Zealand and Australia reported lower rates compared with the overall mean. Age-standardised rates were very low for most countries (lower than five per 1 million person-years in 89.5% of countries), resulting in small absolute differences among countries. As noted with the proportions analysis, trend analysis showed an overall 60% increase in hypoglycaemia-related deaths until 2010 and stable rate trends onwards; rising rates were particularly evident for Brazil, Chile and the USA.

**Conclusions/interpretation:**

Most countries in South America, Central America and the Caribbean showed the highest proportions of diabetes-related deaths attributable to hypoglycaemia and the highest rates of hypoglycaemia-related deaths. Between 2000 and 2014, rising trends were observed in Brazil, Chile and the USA for both rates and proportions of hypoglycaemia-related death, and in Argentina and Japan for proportions only. Further studies are required to unravel the contribution of clinical and socioeconomic factors, difference in diabetes prevalence and heterogeneity of death certification in determining lower rates and proportions of hypoglycaemia-related deaths in high-income countries in Europe, North America and Asia.

**Data availability:**

Data used for these analyses are available at 10.17632/ndp52fbz8r.1

**Electronic supplementary material:**

The online version of this article (10.1007/s00125-018-4626-y) contains peer-reviewed but unedited supplementary material, which is available to authorised users.

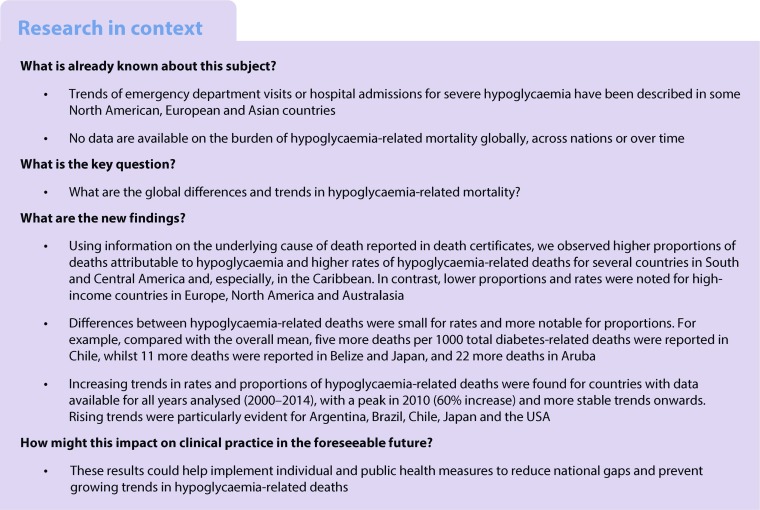



## Introduction

The global prevalence of diabetes is steadily increasing, with an estimated 422 million adults having diabetes in 2014 [1]. Diabetes is an important cause of premature death associated with a twofold higher risk of cardiovascular mortality [2, 3]. In randomised controlled trials, intensive glucose control reduces microvascular and, to some extent, macrovascular complications [4]. Targeting better glucose control, however, results in higher rates of hypoglycaemia and this has a negative impact on quality of life and prevents maintenance of a good glycaemic control [5, 6]. Severe hypoglycaemia can also result in short-term complications, including injuries, car accidents and fatal cardiovascular events due to an increased risk of arrhythmias [7–9], particularly in elderly individuals and those at high risk, or with a history of, cardiovascular disease [10–13].

Large observational studies have reported increasing trends of severe hypoglycaemia, resulting in emergency visits or hospitalisation, in the USA [14, 15], Canada [16], England [17] and Japan [18], possibly related to the rising prevalence of diabetes in older people; yet, the global burden of hypoglycaemia-related mortality is unknown. Using information on the underlying cause of death reported in death certificates, we aimed to comprehensively investigate the global burden, national differences and temporal trends of deaths reporting hypoglycaemia as the underlying cause.

## Methods

### Data sources

**Fig. 1:**
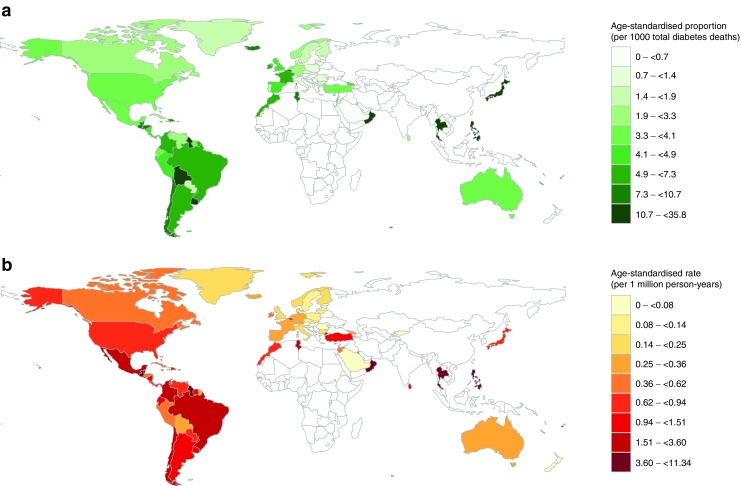
Choropleth maps of age-standardised proportions (**a**) and rates (**b**) of hypoglycaemia-related deaths. White areas indicate countries for which data were not available

We used two main sources of information (electronic supplementary material [ESM] Fig. 1). First, we extracted mortality data for the years 2000 to 2014 from the WHO mortality database, which collates country-specific statistics on cause of death by year, sex and age (who.int/healthinfo/statistics/mortality_rawdata/en, accessed 9 February 2017). The underlying cause of death is recorded in accordance with the principles of the ICD and is defined as the cause that initiated the series of events resulting in death. We used ICD-10 (www.who.int/classifications/icd/en/), with detailed codes (4th character, ICD-104) to allow collection of data on deaths reporting hypoglycaemia as the underlying cause (i.e. E160, drug-induced hypoglycaemia without coma; E161, other hypoglycaemia; E162, hypoglycaemia, unspecified). For the same countries with available ICD-104 codes, we also collected data on death certificates reporting diabetes as the underlying cause of death (E10–E14 codes). When countries reported missing data for one or more years, we searched for official websites of each country; we were able to extract further information for Canada (year 2012) (www5.statcan.gc.ca/cansim/a26?lang=eng&retrLang=eng&id=1020524&&pattern=&stByVal=1&p1=1&p2=35&tabMode=dataTable&csid=, accessed 20 February 2017). Second, for countries with available death data (ESM Table 1), we accessed the United Nations website and extracted mid-year population estimates by year, sex and age (data.un.org, accessed 17 February 2017). We performed this study in accordance with Guidelines for Accurate and Transparent Health Estimates Reporting (GATHER).

### Data analysis

After unifying age groups across mortality and population databases (0–4, 5–9, 10–14 years of age, etc., up to 80–84 and 85 or more years of age), we performed two analyses. In the first, we estimated crude and age-standardised proportions of hypoglycaemia-related deaths using the number of deaths reporting hypoglycaemia as the underlying cause as numerator and the sum of deaths for hypoglycaemia and for diabetes (total diabetes deaths) as denominator. Direct standardisation used overall diabetes- and hypoglycaemia-related deaths obtained from countries with complete data between 2000 and 2014 (31 countries; ESM Table 1); proportions were reported per 1000 total diabetes deaths. In addition, we calculated crude and age-standardised rates of hypoglycaemia-related deaths using the same numerator and mid-year populations as exposure (denominator). Direct standardisation used WHO standard population and rates were reported per 1 million person-years [19]. Using crude and standardised (adjusted) number of hypoglycaemia-related deaths, we performed logistic and Poisson regressions to assess crude and age-standardised trends for proportions and rates, respectively. To avoid bias, overall trends were calculated using only data from countries with all years analysed available and were reported as ORs (which are similar to prevalence ratios for rare events [20]) and incidence rate ratios for proportions and rates, respectively; we considered year as factor variable and used the first calendar year (2000) as reference. We used the same method to compare crude and standardised proportions and rates across countries, taking the overall crude and standardised estimates obtained by combining all countries as reference. All analyses were performed with Stata MP (StataCorp. 2015. Stata Statistical Software: Release 14.1 College Station; TX, USA); choropleth maps and heat plots were obtained in R (R Core Team 2017, version 3.2.3; http://www.R-project.org/) with packages choroplethr (https://cran.r-project.org/web/packages/choroplethr/index.html, accessed 3 March 2017) and ggplot2 (http://ggplot2.org/, accessed 3 March 2017), respectively. Results are reported with 95% CIs and a *p* value <0.05 was considered statistically significant.

## Results

### Proportions of hypoglycaemia-related death

Data on hypoglycaemia- and diabetes-related deaths were available for 109 countries and were complete for 31 countries (28.4%) (ESM Table 1). The age-standardised proportion per 1000 total diabetes deaths was <10 in 91 (83.5%) countries (Fig. 1a and ESM Figs 2–4), ranging from 0.11 (95% CI 0.01, 0.69) in Croatia to 283.1 (95% CI 40.5, 805.7) in Kiribati (ESM Table 2 and ESM Fig. 3). The standardised overall proportion combining all countries was 4.49 (95% CI 4.44, 4.55) per 1000 total diabetes deaths.

Among the countries with complete data, Aruba, Belize and Saint Vincent and the Grenadines displayed age-standardised proportions of hypoglycaemia-related deaths that were two to six times higher than the reference estimate combining all countries (ESM Fig. 5). Large South American countries showed higher proportions compared with the overall mean; the age-standardised OR (95% CI) was 1.09 (1.06, 1.13) for Brazil, 1.37 (1.28, 1.47) for Argentina and 2.10 (1.92, 2.30) for Chile (ESM Fig. 5), with corresponding proportion differences of 0.42 (0.25, 0.59), 1.66 (1.23, 2.10) and 4.94 (4.10, 5.78) per 1000 total diabetes deaths, respectively (ESM Table 3). In contrast, lower proportions were found for Mexico (0.82 per 1000 total diabetes deaths; 95% CI 0.79, 0.85), Puerto Rico (0.72 per 1000 total diabetes deaths; 95% CI 0.60, 0.85) and Paraguay (0.41 per 1000 total diabetes deaths; 95% CI 0.30, 0.55) (ESM Fig. 5). The USA and several European countries with complete data (Croatia, Czech Republic, Finland, Germany, Hungary, Norway, Poland, Romania and Sweden) also reported lower proportions, with age-standardised ORs ranging from 0.88 per 1000 total diabetes deaths (95% CI 0.85, 0.91) for the USA to 0.02 (0.01, 0.10) for Croatia, corresponding to proportion differences of −0.55 (−0.68, −0.42) and −4.39 (−4.55, −4.22) per 1000 total diabetes deaths, respectively (ESM Fig. 5 and ESM Table 3).

In countries with data unavailable for 1 or 2 years, age-standardised ORs and proportion differences (compared with estimated mean) were higher for Japan, Uruguay, Belgium, Colombia and France and lower for Cuba, Venezuela, Canada, Australia, New Zealand, Austria, Denmark, Switzerland and the Netherlands (ESM Fig. 5 and ESM Table 3). For countries with data unavailable for three or more years, higher proportions were observed for Thailand, the Philippines and Guatemala and lower for Turkey, Portugal and Italy (ESM Table 3 and ESM Table 4).

**Fig. 2:**
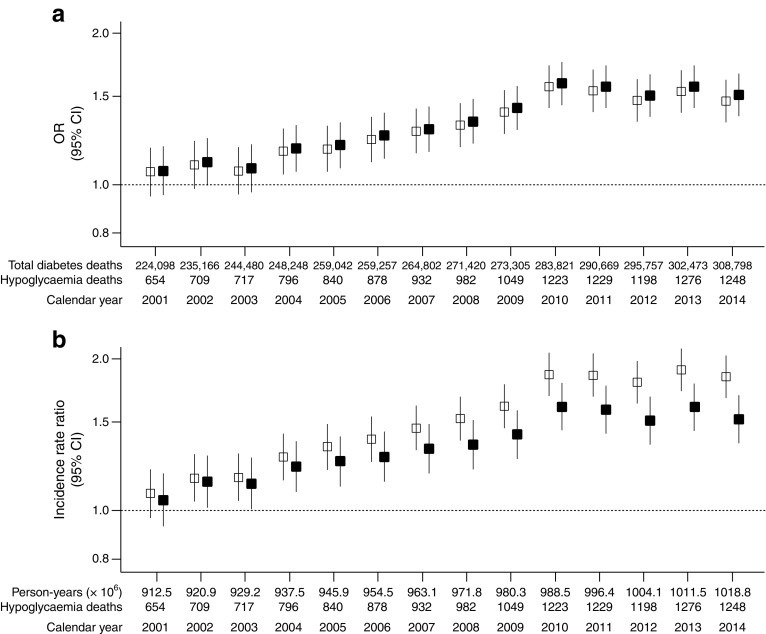
Overall trends in crude and age-standardised proportions and rates of hypoglycaemia-related deaths for countries with complete data between 2000 and 2014. Trends for (**a**) proportions (ORs) and (**b**) rates (incidence rate ratios) of hypoglycaemia-related deaths are shown compared with year 2000 (reference [1.0]; dotted line). In 2000, there were 600 hypoglycaemia-related deaths and 218,011 total diabetes deaths per 903.84 × 10^6^ person-years. Data presented as estimate ± 95% CI. White squares, crude estimates; black squares, age-standardised estimates

**Fig. 3:**
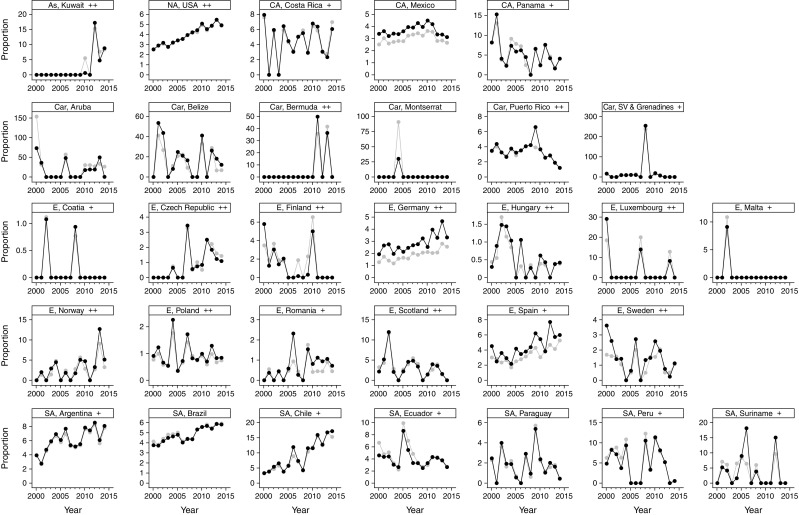
Country-specific trends in crude and age-standardised proportions of hypoglycaemia-related deaths. Trends in hypoglycaemia-related deaths are shown for countries with data available for all years analysed (2000–2014); proportions are shown per 1000 total diabetes-related deaths. Grey circles, crude estimates; black circles, age-standardised estimates. Countries are arranged by geographical region: As, Asia; NA, North America; CA, Central America; Car, Caribbean; E, Europe; SA, South America. Sociodemographic index: +, high–middle; ++, high; no sign, data not available. SV, Saint Vincent

Between 2000 and 2014, crude age-specific proportions changed negligibly: the highest were found in individuals younger than 10 years old, with up to 350 hypoglycaemia-related deaths per 1000 total diabetes deaths; for all individuals over 25 years of age, proportions were lower than 18 per 1000 total diabetes deaths, with no difference between young, middle-aged and older adults (ESM Fig. 6). The overall trends for countries with complete data showed similar crude and standardised proportions, with an increase until 2010 and a plateau thereafter (Fig. 2a): compared with year 2000, the age-standardised OR (95% CI) was 1.59 (1.44, 1.75) in 2010 and 1.51 (1.37, 1.66) in 2014. Rising trends in crude and age-standardised proportions were observed in Argentina, Brazil, Chile and the USA (Fig. 3): the age-standardised OR (95% CI) for 2014 vs 2000 was 5.20 (2.54, 10.68) for Chile, 2.06 (1.37, 3.10) for Argentina, 1.94 (1.62, 2.32) for the USA and 1.55 (1.27, 1.89) for Brazil; the respective corresponding proportion difference (95% CI) was 13.89 (9.38, 18.39), 4.16 (1.82, 6.50), 2.38 (1.76, 3.00) and 2.06 (1.17, 2.95) per 1000 total diabetes deaths. In Japan, where data were unavailable for 1 year, a rising trend was observed: the age-standardised OR for 2013 vs 2000 was 2.73 (95% CI 2.18, 3.42), corresponding to a proportion difference of 14.02 (95% CI 11.13, 16.91) per 1000 total diabetes deaths (ESM Fig. 7).

### Rates of hypoglycaemia-related death

Data on mortality and mid-year populations were available for 105 countries and were complete for 30 countries (28.6%) (ESM Table 1). The age-standardised rate was lower than five per 1 million person-years in 94 countries (89.5%) (Fig. 1b and ESM Figs 2–4), ranging from 0.01 (95% CI 0.00, 0.08) in Croatia to 109.9 (95% CI 40.6, 226.0) in Kiribati (ESM Table 2 and ESM Fig. 3). The standardised overall rate combining all countries was 0.79 (95% CI 0.77, 0.80).

Among the countries with complete data, Belize, Saint Vincent and the Grenadines and Aruba displayed age-standardised rates of hypoglycaemia-related deaths that were 9- to 15-times higher than the reference estimate combining all countries (ESM Fig. 5). Argentina, Brazil, Chile, Ecuador, Mexico, Panama and Puerto Rico also showed higher rates: the age-standardised rate ratio (95% CI) ranged from 3.16 (3.05, 3.26) for Mexico to 1.27 (1.17, 1.38) for Argentina (ESM Fig. 5), although corresponding rate differences were small, at 1.69 (1.62, 1.77) and 0.21 (0.13, 0.29) per 1 million person-years, respectively (ESM Table 5). The USA and virtually all European countries with complete data (Croatia, Czech Republic, Finland, Germany, Hungary, Norway, Poland, Romania, Sweden, Scotland and Spain) displayed lower rates when compared with the overall estimate, with age-standardised rate ratios (95% CI) ranging from 0.82 (0.79, 0.85) for the USA to 0.02 (0.00, 0.14) for Croatia, corresponding to absolute differences of −0.14 (−0.17, −0.12) and −0.77 (−0.80, −0.74) per 1 million person-years, respectively (ESM Fig. 5 and ESM Table 5).

Among those countries for which data were unavailable for 1 or 2 years, South American, Central American and Caribbean countries showed higher rates and European countries, Canada, Japan, New Zealand and Australia showed lower rates when compared with the overall estimate (ESM Fig. 5 and ESM Table 5). For countries with data unavailable for three or more years, the age-standardised rate ratios were higher for the Philippines, Thailand, Guatemala and Turkey and lower for Ireland, Portugal and Italy (ESM Tables 5 and 6).

**Fig. 4:**
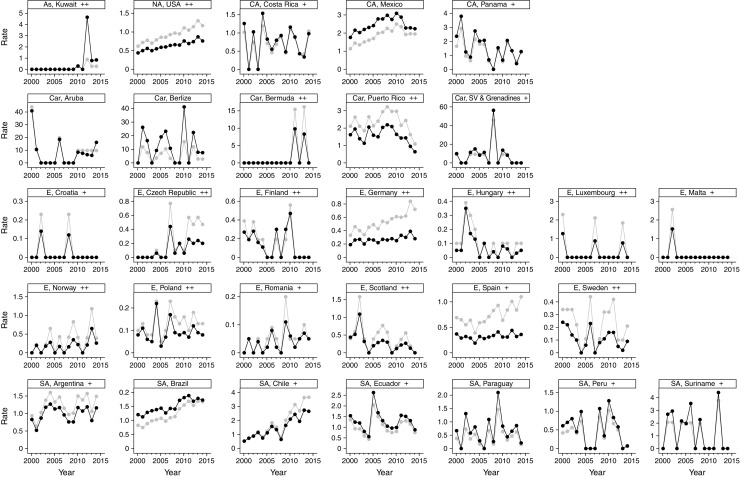
Country-specific trends in crude and age-standardised rates of hypoglycaemia-related deaths. Trends in hypoglycaemia-related deaths are shown for countries with data available for all years analysed (2000–2014); rates are shown per 1 million person-years. Grey circles, crude estimates; black circles, age-standardised estimates. Countries are arranged by geographical region: As, Asia; NA, North America; CA, Central America; Car, Caribbean; E, Europe; SA, South America. Sociodemographic index: +, high–middle; ++ high; no sign, data not available. SV, Saint Vincent

Between 2000 and 2014, crude age-specific rates of hypoglycaemia-related death increased mainly in individuals over the age of 50 years (ESM Fig. 6). In younger people, rates were consistently lower than 1.4 per 1 million person-years but they increased about fourfold in individuals aged 50–75 years and 16-fold in those over the age of 85 years. The overall trend for countries with complete data showed a steady increase until 2010 and a plateau thereafter (Fig. 2b): compared with year 2000, the age-standardised rate ratio (95% CI) peaked in 2010 at 1.61 (1.44, 1.79) and plateaued thereafter (rate ratio in 2014: 1.52 [1.36, 1.69]). Rising trends in crude and age-standardised rates were observed in Brazil, Chile and the USA (Fig. 4): the age-standardised rate ratio (95% CI) for 2014 vs 2000 was 5.05 (2.38, 10.68) for Chile, 1.74 (1.41, 2.17) for the USA and 1.42 (1.20, 1.69) for Brazil, with respective corresponding differences of 2.13 (1.29, 2.97), 0.32 (0.20, 0.45) and 0.52 (0.27, 0.76) per 1 million person-years. In some countries where data were unavailable for 1 or 2 years, including El Salvador, Japan, Uruguay and Venezuela (ESM Fig. 8), an increasing trend was also observed.

## Discussion

These results indicate higher proportions of deaths reporting hypoglycaemia as the underlying cause for most Caribbean, South American and Central American countries (ESM Table 7). Proportions were also higher in the Philippines, Thailand and Japan and were lower in Australia, New Zealand, the USA, Canada and most European countries. When compared with the overall mean, estimates show about five more hypoglycaemia-related deaths per 1000 total diabetes deaths in Chile, six in Belgium and Uruguay, eight in Barbados, nine in Guadeloupe, 11 in Belize and Japan and 22 in Aruba. The results for rates mostly mirrored those for proportions, except in Japan which showed lower rates but higher proportions. However, differences in rates were very small because absolute rates were very low for most countries (lower than five per 1 million person-years in about 90% of countries). For countries with data available for the entire period of observation, trend analysis showed an overall 60% increase in both proportions and rates until 2010, compared with 2000, and more stable levels thereafter. Increasing rates were particularly evident for Brazil, Chile and the USA, although absolute differences were small. Conversely, rising trends in proportions for the same three countries plus Argentina translated into about 14 more hypoglycaemia-related deaths per 1000 total diabetes deaths for Chile, four for Argentina and two for the USA and Brazil, comparing 2014 with 2000. Fourteen more deaths occurred in Japan, comparing 2013 with 2000.

Differences between countries could be explained by pathophysiological differences among individuals of diverse ethnicity, cultural dissimilarities or variations in diabetes management. Observational analyses conducted in North America reported higher rates of severe hypoglycaemia and hospital admission for hypoglycaemia in African-Americans, compared with other ethnicities [14, 21–24]. Moreover, a recent study in England has confirmed a different risk of hypoglycaemia in people with diabetes of diverse ethnicity, reporting a 60% higher risk of hospital admission for severe hypoglycaemia in people of Caribbean descent, compared with those of European descent, accounting for socioeconomic differences [25]. In these studies, individuals of different ethnicity shared the same healthcare system, so the increased risk of hypoglycaemia would likely be related to intrinsic, biological differences more than external factors. These results would suggest pathophysiological differences exist across ethnicities, making Caribbean populations more vulnerable to severe hypoglycaemia. Such a hypothesis is further supported by the observation that countries reporting higher rates have high, high–middle and middle sociodemographic development (measured as Socio-demographic Index, SDI) (ESM Table 7) [26]. At the same time, we observed higher proportions of hypoglycaemia-related deaths in countries with both high–middle and middle SDI. The different SDI for these countries would not support social and economic factors as determinants of a higher risk.

Of note, 21 out of 26 European countries (80.7%) with lower rates and 16 out of 21 (76.2%) with lower proportions, have high SDI; similarly, Australia, Canada, the USA and New Zealand have high SDI and lower rates and proportions. These observations, along with acknowledgement of the high cost of measures aimed at reducing hypoglycaemia (i.e. structured education, psychological support, glucose self-monitoring, expensive glucose-lowering drugs associated with a lower risk of hypoglycaemia compared with sulfonylureas and insulin) [27–30], would point to the opposite hypothesis, underlying the relevance of socioeconomic status as a determinant of country differences.

The higher rates displayed in Central and South American countries confirm the findings from a recent observational analysis (Hypoglycaemia Assessment Tool study), which reported a twofold greater risk of severe hypoglycaemia in individuals living in Argentina and Mexico [31]. On the other hand, our results for Asian countries were mixed and fewer data on the risk of hypoglycaemia in Asian populations are currently available. Our results for Japan, indicating 11 more hypoglycaemia-related deaths per 1000 total diabetes deaths compared with the overall estimate, are particularly interesting. As for Caribbean populations, pathophysiological, socioeconomic and demographic differences could explain the conflicting results for Asian countries.

Using data from countries with all years analysed (2000–2014) available, we observed increasing trends of rates and proportions until 2010 followed by more stable estimates until 2014. Rising trends in severe hypoglycaemia (resulting in emergency department visit or hospital admissions) have been reported in recent years for several high-SDI countries (USA, Canada, Japan, England) [14–18]. Most of these studies revealed a peak (2007 in the USA [14], 2006 in Canada and 2010 in Japan and England) followed by more stable trends. The acknowledgement of the potential harm of hypoglycaemia in individuals treated intensively, a greater attention towards hypoglycaemia, the recommendations of personalised targets by international guidelines and the availability of new drugs associated with a lower risk of hypoglycaemia in high-income countries could explain the more stable trends of severe hypoglycaemia observed in the previous studies and our results [32]. However, together with Argentina, Brazil and Chile, rising trends for rates and proportions were also observed in high-SDI countries such as the USA and Japan. An unnecessary intensive use of glucose-lowering therapies for older individuals with diabetes and multimorbidity has been reported in the USA and could explain our results [33, 34]. However, the reasons for increasing trends in Japan require further investigation.

Differences in the availability of essential drugs and supplies for diabetes treatment might account for some of the variance we observed among countries. The only available sulfonylurea in 60% of lower- and middle-income countries is glibenclamide [35], which has been replaced by gliclazide in the WHO list of essential medicines because of its significantly lower risk of hypoglycaemia [36]. Glucagon, which is potentially life-saving in severe episodes of hypoglycaemia, is fully available in 80% of high-income countries and 41% of middle-income countries [37]. The availability of newer and more expensive glucose-lowering drugs with a low risk of hypoglycaemia is also inferior in middle-income compared with high-income countries (7–13% vs 47–52%) [37] and access to blood glucose meters and test strips, indispensable for reducing the risk of hypoglycaemia in insulin-treated individuals, is 50% lower in middle-income compared with high-income countries [37]. These observations further underline the relationship between socioeconomic factors and risk of hypoglycaemia and suggest that different strategies for different countries are needed to reduce the risk of potentially fatal hypoglycaemic episodes. Country-level, socioeconomic determinants should be first addressed in middle-income countries, while strategies focusing on individuals should be the priority in high-income countries.

The use of information gained from death certificates is a well-established approach to describe the spatiotemporal burden of diseases. Invaluable data have been obtained in this way, allowing clarification of the global burden of several medical conditions, including infections, cancer and cardiovascular, metabolic, digestive, respiratory, neurological and mental health disorders [38]. Such data are instrumental for measuring population health, identifying inequalities and possible risk factors and triggering public health interventions. To our knowledge, this is the first study to estimate the burden of hypoglycaemia-related deaths using this approach.

Our research has some limitations. First, to identify diabetes- and hypoglycaemia-related deaths, we relied on the WHO mortality database; this, in principle, is the optimal source of mortality data as the data result from a medically certified cause of death [39]. Moreover, in instances where physicians report an incorrect sequence of causal relationships for a disease, the WHO uses a series of rules to select the underlying cause [40]. When mortality is investigated using death certificates, it is commonly assumed that the probability of recording the underlying cause of death for a specific disease remains constant over time and is not different across countries (i.e. homogeneous outcome definition and assessment). However, differences in the identification or definition of medical conditions, or in their order in a causal sequence, are possible. This, along with errors in certificates and misinterpretation of ICD rules for selection of the underlying cause, could result in heterogeneous definition and assessment of the underlying cause of death for both diabetes and hypoglycaemia. This heterogeneity is a well-known problem of all epidemiological analyses based on death certificates and could certainly have influenced our results, although to what extent is difficult to quantify. Some studies have reported factors associated with a higher likelihood of reporting diabetes as the underlying cause of death (longstanding disease, insulin treatment, fewer comorbidities) [41], possible underestimations of diabetes deaths using certificates [42], incorrect reporting of causal sequences [43] and international differences in the accuracy of certificates [44, 45]. However, the use of death certificates only to detail the global burden of diabetes-related mortality has been previously adopted [46]. Furthermore, it should be noted that identifying a unique underlying cause of death is not always straightforward, particularly in elderly individuals with multiple morbidities. In accordance with the ICD principles, death is attributed to a single underlying cause, although we recognise that concomitant conditions may contribute to the risk of death in individuals with diabetes (i.e. cardiovascular disease).

Differently from diabetes-related deaths, this is the first analysis specifically using hypoglycaemia as the underlying cause documented in death certificates. We cannot exclude the possibility that instances of hypoglycaemia as the ‘true’ reason leading to death have been coded as diabetes-related deaths, although diabetes can be associated with far more frequent and heterogeneous causes of death compared with hypoglycaemia. Similarly, it is possible that some instances of hypoglycaemia and coma have been coded as ‘diabetes mellitus with coma’. The ICD defines the underlying cause of death as the disease or injury that initiated the train of morbid events leading directly to death, or the circumstances of the accident or violence that produced the fatal injury [40]. This, together with the essential role of glucose measurement in the diagnosis of hypoglycaemia (particularly in fatal cases), leads us to believe death certificates that report hypoglycaemia as being the underlying cause in most (if not all) instances when hypoglycaemia was the ‘true’ cause leading directly to death. However, the above epidemiological and clinical considerations should be carefully considered when interpreting our results, in accordance with WHO recommendations.

A second limitation arises because some countries did not report mortality data to the WHO or data were not standardised according to the ICD coding process, meaning that information for some countries and years were not available. We extensively searched mortality data for years not reported in the WHO database, yet we were able to obtain information only for Canada for the year 2012. Third, the coverage of deaths could be incomplete in some countries. Fourth, overall trends were calculated using data from countries with all years analysed (2000–2014) available; these countries showed higher incomes compared with those missing data for one or more years, indicating that increasing trends are mainly representative of hypoglycaemia-related mortality in developed countries. Fifth, we could not differentiate between type 1 and type 2 diabetes. The risk of severe hypoglycaemia and hypoglycaemia-related mortality is different between the two phenotypes and their prevalence varies across countries [47]. While in type 1 diabetes severe hypoglycaemia is more likely to be the underlying cause of death, in type 2 diabetes a higher prevalence of cardiovascular disease could contribute to an increased risk of fatal hypoglycaemia. Moreover, type 1 diabetes is only treated by insulin whereas for type 2 diabetes a wide range of glucose-lowering medications is available, each being associated with a different risk of hypoglycaemia. Sixth, we did not account for the change in diabetes prevalence across countries and over time; however, we considered total diabetes deaths which should be, in part, indicative of diabetes prevalence. Last, some estimates for rates and proportions have wide imprecision (CIs) as a result of few events; we opted to perform a complete analysis and report trends stratified by countries with complete data or missing for one or more years instead of selecting a minimum number of events for inclusion in the analyses.

In conclusion, in this study we observed national differences in the burden of deaths related to hypoglycaemia. Most South American, Central American and Caribbean countries reported the highest rates with Brazil and Chile also demonstrating an increasing trend between 2000 and 2014; however, absolute rate differences comparing countries and over time were small. Conversely, differences were more notable for the proportions of deaths attributable to hypoglycaemia. Although the geographical pattern was similar, with the highest proportions for South American, Central American and Caribbean countries and increasing trends for Argentina, Brazil and Chile, we also found rising trends for the USA and, particularly, Japan. Further studies are required to quantify the impact of differences in diabetes prevalence and certificate coding, through time and between countries, in determining our results. This complementary information will disentangle the complex syndemic of biological and socioeconomic factors to reduce national differences and prevent rising trends in hypoglycaemia-related deaths.

## Electronic supplementary material

ESM (PDF 3.99 mb)

## Data Availability

Databases are available at WHO (http://www.who.int/healthinfo/statistics/mortality_rawdata/en) and UN (data.un.org) websites. Summary data used for these analyses are available at 10.17632/ndp52fbz8r.1. Statistical codes are available from the corresponding author (FZ).

## Abbreviation

SDI: Socio-demographic Index
