# Supplementary material for: Global burden of hypoglycaemia-related mortality in 109 countries, from 2000 to 2014: an analysis of death certificates
Source: Diabetologia. 2018 May 1;61(7):1592–602. doi: 10.1007/s00125-018-4626-y (PMC6438613; doi:10.1007/s00125-018-4626-y)
Supplement: Supplementary file 1 — (PDF 3.99 mb) [file 125_2018_4626_MOESM1_ESM.pdf]

Electronic Supplementary Material

Global burden of hypoglycaemia-related mortality in 109 countries, from 2000 to 2014: an analysis of death certificates

Francesco Zaccardi[1], Nafeesa N Dhalwani[1], David R Webb[1], Melanie J Davies[1], Kamlesh Khunti[1]

[1] Diabetes Research Centre, University of Leicester, Leicester, UK

**ESM Table 1**	Unavailable data for mid-year population and hypoglycaemia death, by country and year	2
**ESM Table 2**	Crude and age-standardised proportions and rates with 95% confidence intervals, by country	4
**ESM Table 3**	Crude and age-standardised proportion differences compared to the overall estimate, by country	6
**ESM Table 4**	Crude and age-standardised odds ratios compared to the overall estimate, for countries with three or more years unavailable	7
**ESM Table 5**	Crude and age-standardised rate differences compared to the overall estimate, by country	8
**ESM Table 6**	Crude and age-standardised rate ratios compared to the overall estimate, for countries with three or more years unavailable	9
**ESM Table 7**	Age-standardised proportion and rate differences compared to their respective overall estimates, by geographical area and socio-demographic development	10
**ESM Figure 1**	Flow-diagram of data search process	11
**ESM Figure 2**	Country-specific crude and age-standardised proportion and rate	12
**ESM Figure 3**	Crude and age-standardised proportions and rates, by country	13
**ESM Figure 4**	Year-specific crude and age-standardised proportions and rates, by country	14
**ESM Figure 5**	Age-standardised odds and rate ratios	15
**ESM Figure 6**	Age-specific proportions and rates, by year	16
**ESM Figure 7**	Trends of crude and age-standardised proportions for countries with data missing for one or two years	17
**ESM Figure 8**	Trends of crude and age-standardised rates for countries with data missing for one or two years	18

ESM Table 1: Unavailable data (✖) for mid-year population and hypoglycaemia death, by country and year

	Population data by age groups – years 2000s															Diabetes and hypoglycaemia deaths by age groups – years 2000s														
Country	00	01	02	03	04	05	06	07	08	09	10	11	12	13	14	00	01	02	03	04	05	06	07	08	09	10	11	12	13	14
Anguilla	✖		✖	✖	✖	✖	✖	✖	✖	✖	✖	✖	✖	✖	✖		✖						✖							
Antigua and Barbuda																										✖	✖			
Argentina																														
Armenia																✖	✖	✖	✖	✖	✖	✖	✖	✖	✖	✖	✖	✖	✖	
Aruba																														
Australia																					✖									
Austria																✖	✖													
Bahamas																														✖
Barbados																														✖
Belgium																														✖
Belize																														
Bermuda																														
Bolivia																				✖	✖	✖	✖	✖	✖	✖	✖	✖	✖	✖
Brazil																														
British Virgin Islands	✖		✖	✖	✖	✖	✖	✖	✖	✖		✖	✖	✖	✖						✖		✖				✖	✖	✖	✖
Bulgaria																✖	✖	✖	✖	✖	✖	✖	✖	✖	✖	✖	✖	✖		✖
Canada																												@	✖	✖
Cayman Islands	✖	✖	✖	✖	✖	✖		✖																			✖	✖		✖
Chile																														
Colombia																														✖
Costa Rica																														
Croatia																														
Cuba																✖														
Cyprus																✖	✖	✖	✖											✖
Czech Republic																														
Denmark																													✖	✖
Dominica	✖		✖	✖	✖	✖		✖	✖	✖	✖	✖	✖	✖	✖	✖														
Dominican Republic																													✖	✖
Ecuador																														
El Salvador																														✖
Estonia																✖	✖	✖	✖	✖	✖	✖	✖	✖					✖	
Fiji																✖										✖			✖	✖
Finland																														
France																														✖
French Guiana																✖														✖
Georgia																✖	✖	✖	✖					✖						
Germany																														
Grenada																✖														
Guadeloupe																														✖
Guatemala																✖	✖	✖	✖	✖										
Guyana																✖													✖	✖
Haiti																✖					✖	✖	✖	✖	✖	✖	✖	✖	✖	✖
Honduras																✖	✖	✖	✖	✖	✖	✖	✖							✖
Hong Kong SAR																✖														✖
Hungary																														
Iceland																										✖	✖	✖	✖	✖
Ireland																✖	✖	✖	✖	✖	✖	✖								✖
Israel																														✖
Italy																✖	✖	✖		✖	✖								✖	✖
Jamaica																							✖	✖				✖	✖	✖
Japan																														✖
Jordan																✖	✖	✖	✖	✖	✖	✖	✖					✖	✖	✖
Kiribati																		✖	✖	✖	✖	✖	✖	✖	✖	✖	✖	✖	✖	✖
Kuwait																														
Kyrgyzstan																														✖
Latvia																✖	✖	✖	✖	✖	✖	✖	✖							
Lithuania																✖														
Luxembourg																														

ESM Table 1 (cont'd): Unavailable data (×) for population and hypoglycaemia deaths, by country and year

	Population data by age groups – years 2000s															Diabetes and hypoglycaemia deaths by age groups – years 2000s														
Country	00	01	02	03	04	05	06	07	08	09	10	11	12	13	14	00	01	02	03	04	05	06	07	08	09	10	11	12	13	14
Maldives																×	×	×	×	×	×	×			×			×	×	×
Malta																														
Martinique																												×		×
Mauritius																×	×	×	×	×										
Mayotte																×	×	×	×	×	×	×	×	×	×	×	×			×
Mexico																														
Montserrat	×		×	×	×	×		×	×	×	×		×	×	×															
Morocco																×	×	×	×	×	×	×	×						×	×
Netherlands																														×
Netherlands Antilles	×	×	×	×	×	×	×	×	×	×	×	×	×	×	×		×	×	×	×	×	×	×	×	×	×	×	×	×	×
New Zealand																													×	×
Nicaragua																														×
Norway																														
Occupied Palestinian Territory																×	×	×	×	×	×	×	×			×	×	×	×	×
Oman																×	×	×	×	×	×	×	×	×	×		×	×	×	×
Panama																														
Paraguay																														
Peru																														
Philippines																				×	×			×				×	×	×
Poland																														
Portugal																×	×			×	×	×								
Puerto Rico																														
Republic of Moldova																×	×	×	×	×	×	×								
Reunion																×														×
Rodrigues	×	×	×	×	×	×	×	×	×	×	×	×	×	×	×	×	×	×	×	×										
Romania																														
Saint Kitts and Nevis		×	×	×	×	×	×	×	×	×	×	×	×	×	×														×	×
Saint Lucia																							×							
Saint Pierre and Miquelon	×	×	×	×	×	×		×	×	×	×	×	×	×	×	×	×	×	×	×			×			×	×	×	×	×
Saint Vincent and the Grenadines																														
Saudi Arabia																×	×	×	×	×	×	×	×	×		×	×	×	×	×
Serbia and Montenegro, Former	×	×	×	×	×	×	×	×	×	×	×	×	×	×	×				×	×	×	×	×	×	×	×	×	×	×	×
Singapore																×	×	×	×	×	×	×	×	×	×	×	×			
Slovakia																×	×	×	×	×	×	×	×	×	×		×			
Spain																														
Sri Lanka																×	×	×	×	×	×		×	×	×	×	×	×	×	×
Suriname																														
Sweden																														
Switzerland																														×
Thailand																	×						×	×	×	×	×	×	×	×
Trinidad and Tobago																											×	×	×	×
Tunisia																×	×	×	×	×	×	×	×	×		×	×	×		×
Turkey																×	×	×	×	×	×	×	×	×						×
Turks and Caicos Islands	×		×	×	×	×	×	×	×	×	×	×	×	×	×								×			×	×	×		
*United Kingdom (UK)																×														×
UK, England & Wales																×														
UK, Northern Ireland																×														×
UK, Scotland																														
United States of America																														
Uruguay																											×			
Venezuela																														×
Virgin Islands (USA)																													×	×

* Excluded from the calculation of the overall rate and proportion estimates (used England & Wales, Northern Ireland, and Scotland)
Countries are sorted in alphabetical order
@ http://www5.statcan.gc.ca/cansim/a26?lang=eng&retrLang=eng&id=1020524&&pattern=&stByVal=1&p1=1&p2=35&tabMode=dataTable&csid=

ESM Table 2: Crude and age-standardised proportions and rates with 95% confidence intervals, by country

Country	Total years available		Hypo deaths*	Proportion (95% CI) per 1000 total diabetes deaths		Rate (95% CI) per 1 million person-years	
	Proportion	Rate		Crude	Standardised	Crude	Standardised
Anguilla	13	0	0/-	0.00 (0.00-47.38)	0.00 (0.00-53.37)	-	-
Antigua and Barbuda	13	13	2	3.32 (0.40-11.95)	3.16 (0.37-11.58)	1.83 (0.22-6.62)	1.76 (0.20-6.62)
Argentina	15	15	744	5.87 (5.45-6.30)	6.16 (5.71-6.63)	1.24 (1.15-1.33)	1.00 (0.92-1.07)
Armenia	1	1	0	0.00 (0.00-3.09)	0.00 (0.00-6.28)	0.00 (0.00-1.23)	0.00 (0.00-1.24)
Aruba	15	15	12	23.48 (12.19-40.66)	26.42 (12.78-47.79)	8.05 (4.16-14.05)	7.44 (3.77-13.21)
Australia	14	14	153	2.86 (2.43-3.35)	3.34 (2.77-4.00)	0.52 (0.44-0.60)	0.33 (0.28-0.39)
Austria	13	13	5	0.12 (0.04-0.29)	0.28 (0.07-0.78)	0.05 (0.01-0.11)	0.03 (0.01-0.08)
Bahamas	14	14	7	5.17 (2.08-10.61)	4.93 (1.90-10.08)	1.48 (0.59-3.04)	1.75 (0.70-3.57)
Barbados	14	14	39	13.04 (9.28-17.78)	12.85 (8.89-18.13)	10.10 (7.18-13.80)	6.19 (4.36-8.63)
Belgium	14	14	174	7.48 (6.41-8.67)	10.17 (8.50-12.09)	1.16 (1.00-1.35)	0.62 (0.53-0.73)
Belize	15	15	23	13.38 (8.50-20.01)	15.88 (9.62-24.23)	5.13 (3.25-7.69)	11.34 (7.10-16.94)
Bermuda	15	15	2	5.63 (0.68-20.20)	6.62 (0.72-25.56)	2.11 (0.26-7.61)	1.38 (0.17-5.99)
Bolivia	4	4	7	10.04 (4.05-20.58)	11.12 (3.53-24.49)	0.20 (0.08-0.42)	0.32 (0.12-0.66)
Brazil	15	15	3563	5.04 (4.87-5.20)	4.91 (4.75-5.08)	1.24 (1.20-1.28)	1.54 (1.49-1.59)
British Virgin Islands	9	2	0	0.00 (0.00-69.78)	0.00 (0.00-71.36)	0.00 (0.00-75.98)	0.00 (0.00-94.60)
Bulgaria	1	1	3	2.15 (0.44-6.27)	1.72 (0.35-8.29)	0.41 (0.09-1.21)	0.18 (0.04-0.78)
Canada	13	13	266	2.78 (2.45-3.13)	3.14 (2.74-3.58)	0.63 (0.55-0.71)	0.39 (0.34-0.44)
Cayman Islands	12	5	0	0.00 (0.00-48.63)	0.00 (0.00-76.25)	0.00 (0.00-14.21)	0.00 (0.00-55.84)
Chile	15	15	461	9.05 (8.24-9.91)	9.43 (8.56-10.38)	1.87 (1.70-2.05)	1.58 (1.44-1.73)
Colombia	14	14	728	7.33 (6.80-7.88)	7.22 (6.70-7.78)	1.18 (1.10-1.27)	1.63 (1.51-1.75)
Costa Rica	15	15	44	4.33 (3.15-5.81)	4.45 (3.22-6.00)	0.67 (0.49-0.90)	0.74 (0.54-1.00)
Croatia	15	15	2	0.12 (0.01-0.42)	0.11 (0.01-0.69)	0.03 (0.00-0.11)	0.01 (0.00-0.08)
Cuba	14	14	79	2.69 (2.13-3.36)	2.77 (2.18-3.49)	0.50 (0.40-0.62)	0.38 (0.30-0.48)
Cyprus	10	10	6	1.75 (0.64-3.80)	1.41 (0.45-7.73)	0.56 (0.20-1.21)	0.36 (0.13-0.85)
Czech Republic	15	15	35	1.17 (0.82-1.63)	1.22 (0.79-1.86)	0.23 (0.16-0.31)	0.13 (0.09-0.18)
Denmark	13	13	25	1.44 (0.93-2.12)	1.47 (0.93-2.29)	0.35 (0.23-0.52)	0.18 (0.12-0.29)
Dominica	14	2	5/2	8.14 (2.65-18.90)	9.75 (2.98-25.07)	14.30 (1.73-51.66)	11.16 (1.33-44.51)
Dominican Republic	13	13	119	7.83 (6.49-9.36)	7.56 (6.14-9.16)	0.98 (0.81-1.17)	1.27 (1.05-1.52)
Ecuador	15	15	224	4.35 (3.80-4.96)	3.97 (3.43-4.55)	1.05 (0.92-1.20)	1.29 (1.12-1.47)
El Salvador	14	14	135	7.79 (6.53-9.21)	7.70 (6.38-9.19)	1.62 (1.35-1.91)	2.00 (1.67-2.37)
Estonia	5	5	0	0.00 (0.00-4.57)	0.00 (0.00-4.13)	0.00 (0.00-0.56)	0.00 (0.00-0.58)
Fiji	11	11	54	4.57 (3.44-5.96)	6.11 (4.03-8.74)	5.88 (4.42-7.67)	10.35 (7.50-13.83)
Finland	15	15	12	1.57 (0.81-2.74)	1.48 (0.74-2.63)	0.15 (0.08-0.26)	0.14 (0.07-0.26)
France	14	14	588	3.77 (3.47-4.09)	4.96 (4.48-5.49)	0.68 (0.63-0.74)	0.35 (0.32-0.38)
French Guiana	13	13	5	16.08 (5.24-37.12)	12.21 (3.31-31.10)	1.79 (0.58-4.19)	2.82 (0.85-6.59)
Georgia	10	10	21	2.78 (1.72-4.25)	4.09 (2.28-6.66)	0.49 (0.30-0.75)	0.34 (0.20-0.53)
Germany	15	15	644	1.86 (1.72-2.01)	2.88 (2.61-3.17)	0.53 (0.49-0.57)	0.27 (0.25-0.30)
Grenada	14	14	8	6.89 (2.98-13.52)	7.24 (3.07-14.98)	5.50 (2.37-10.84)	6.00 (2.51-11.98)
Guadeloupe	14	14	23	12.55 (7.97-18.77)	13.96 (8.55-21.67)	3.65 (2.31-5.47)	2.48 (1.55-3.82)
Guatemala	10	10	327	7.68 (6.87-8.55)	7.25 (6.35-8.22)	2.24 (2.01-2.50)	3.60 (3.21-4.03)
Guyana	12	12	60	12.29 (9.39-15.79)	13.47 (9.78-17.96)	6.69 (5.10-8.61)	11.17 (8.45-14.43)
Haiti	4	4	4	6.92 (1.89-17.62)	6.47 (1.20-17.71)	0.11 (0.03-0.29)	0.19 (0.05-0.50)
Honduras	6	6	14	5.66 (3.10-9.48)	8.20 (3.26-15.71)	0.31 (0.17-0.52)	0.39 (0.21-0.66)
Hong Kong SAR	13	13	37	5.14 (3.62-7.08)	4.92 (3.30-7.24)	0.41 (0.29-0.57)	0.24 (0.17-0.34)
Hungary	15	15	17	0.42 (0.25-0.68)	0.58 (0.31-1.04)	0.11 (0.07-0.18)	0.08 (0.04-0.13)
Iceland	10	10	1	4.29 (0.11-23.68)	9.51 (0.24-47.73)	0.34 (0.01-1.88)	0.31 (0.01-1.76)
Ireland	7	7	18	5.08 (3.01-8.01)	6.93 (3.69-11.75)	0.56 (0.33-0.89)	0.44 (0.26-0.71)
Israel	14	14	116	3.42 (2.83-4.10)	3.87 (2.96-5.07)	1.20 (0.99-1.44)	0.85 (0.70-1.03)
Italy	8	8	213	1.30 (1.13-1.49)	1.36 (1.05-1.81)	0.45 (0.39-0.51)	0.15 (0.13-0.18)
Jamaica	10	10	77	4.46 (3.52-5.57)	4.47 (3.51-5.63)	2.88 (2.27-3.60)	2.80 (2.20-3.53)
Japan	14	14	2868	14.90 (14.36-15.45)	15.13 (14.54-15.75)	1.62 (1.56-1.68)	0.67 (0.65-0.70)
Jordan	4	4	6	1.33 (0.49-2.89)	1.43 (0.37-3.49)	0.24 (0.09-0.51)	0.49 (0.15-1.11)
Kiribati	2	2	8	125.00 (55.54-231.53)	283.13 (40.48-805.71)	46.99 (20.29-92.58)	109.91 (40.62-226.04)
Kuwait	15	15	6	2.13 (0.78-4.63)	1.86 (0.60-4.30)	0.15 (0.05-0.33)	0.59 (0.10-1.58)
Kyrgyzstan	14	14	0	0.00 (0.00-0.80)	0.00 (0.00-2.86)	0.00 (0.00-0.05)	0.00 (0.00-0.07)
Latvia	7	7	2	0.59 (0.07-2.14)	0.78 (0.09-2.77)	0.14 (0.02-0.50)	0.09 (0.01-0.40)
Lithuania	14	14	7	1.68 (0.68-3.46)	1.21 (0.46-2.62)	0.16 (0.06-0.32)	0.13 (0.05-0.29)
Luxembourg	15	15	3	3.54 (0.73-10.30)	2.61 (0.53-9.37)	0.41 (0.09-1.21)	0.19 (0.04-0.75)
Maldives	4	4	3	32.97 (6.85-93.33)	28.00 (5.73-88.14)	2.29 (0.47-6.70)	3.79 (0.78-10.82)
Malta	15	15	1	0.64 (0.02-3.53)	0.46 (0.01-6.03)	0.17 (0.00-0.92)	0.09 (0.00-0.73)
Martinique	13	13	11	6.70 (3.35-11.96)	8.82 (3.86-17.14)	2.15 (1.07-3.84)	1.28 (0.62-2.44)
Mauritius	10	10	56	2.64 (2.00-3.43)	2.68 (1.98-3.59)	4.50 (3.40-5.84)	4.38 (3.30-5.71)
Mayotte	2	2	0	0.00 (0.00-92.51)	0.00 (0.00-126.46)	0.00 (0.00-8.23)	0.00 (0.00-13.53)
Mexico	15	15	3192	3.03 (2.93-3.14)	3.68 (3.55-3.82)	1.87 (1.81-1.94)	2.48 (2.39-2.57)
Montserrat	15	3	1/0	6.94 (0.18-38.08)	10.03 (0.25-66.52)	0.00 (0.00-261.25)	0.00 (0.00-257.82)
Morocco	5	5	105	8.76 (7.17-10.60)	6.86 (5.41-8.55)	0.65 (0.53-0.79)	0.77 (0.63-0.94)
Netherlands	14	14	125	2.62 (2.18-3.12)	2.89 (2.31-3.58)	0.55 (0.45-0.65)	0.29 (0.24-0.35)
Netherlands Antilles	1	0	0/-	0.00 (0.00-115.70)	0.00 (0.00-122.96)	-	-
New Zealand	13	13	5	0.46 (0.15-1.08)	0.57 (0.17-1.42)	0.09 (0.03-0.22)	0.08 (0.02-0.18)
Nicaragua	14	14	58	3.37 (2.56-4.36)	4.16 (3.06-5.48)	0.76 (0.57-0.98)	1.29 (0.97-1.67)
Norway	15	15	25	2.34 (1.52-3.46)	2.83 (1.68-4.44)	0.35 (0.23-0.52)	0.18 (0.11-0.28)
Occupied Palestinian Territory	2	2	0	0.00 (0.00-11.98)	0.00 (0.00-14.91)	0.00 (0.00-0.47)	0.00 (0.00-1.17)
Oman	1	1	7	36.65 (14.86-74.05)	31.67 (7.19-82.77)	2.38 (0.96-4.90)	5.28 (1.91-11.11)
Panama	15	15	66	5.12 (3.96-6.51)	4.88 (3.76-6.25)	1.28 (0.99-1.63)	1.46 (1.12-1.85)

ESM Table 2 (cont'd): Crude and age-standardised proportions and rates, by country

	Total years available		Hypo deaths*	Proportion (95% CI) per 1000 total diabetes deaths		Rate (95% CI) Per 1 million person-years	
Country	Proportion	Rate		Crude	Standardised	Crude	Standardised
Paraguay	15	15	40	1.72 (1.23-2.34)	1.84 (1.30-2.51)	0.45 (0.32-0.61)	0.68 (0.49-0.93)
Peru	15	15	168	4.99 (4.26-5.80)	4.58 (3.88-5.36)	0.40 (0.34-0.46)	0.51 (0.43-0.59)
Philippines	9	9	2503	15.56 (14.96-16.18)	25.96 (24.80-27.15)	3.22 (3.09-3.35)	8.75 (8.38-9.13)
Poland	15	15	77	0.84 (0.66-1.05)	0.96 (0.75-1.21)	0.13 (0.11-0.17)	0.09 (0.07-0.12)
Portugal	10	10	66	1.46 (1.13-1.85)	1.41 (0.99-2.10)	0.63 (0.49-0.80)	0.25 (0.19-0.33)
Puerto Rico	15	15	132	3.14 (2.63-3.72)	3.22 (2.68-3.85)	2.35 (1.97-2.79)	1.57 (1.31-1.88)
Republic of Moldova	8	8	0	0.00 (0.00-1.33)	0.00 (0.00-2.31)	0.00 (0.00-0.11)	0.00 (0.00-0.12)
Reunion	13	13	24	7.82 (5.02-11.61)	8.76 (5.59-13.99)	2.29 (1.47-3.41)	2.29 (1.46-3.41)
Rodrigues	10	0	1/-	2.35 (0.06-13.04)	2.26 (0.06-15.46)	-	-
Romania	15	15	16	0.50 (0.28-0.80)	0.65 (0.35-1.10)	0.05 (0.03-0.08)	0.04 (0.02-0.06)
Saint Kitts and Nevis	13	1	4/0	12.16 (3.32-30.84)	11.48 (3.10-30.34)	0.00 (0.00-91.29)	0.00 (0.00-102.20)
Saint Lucia	14	14	4	2.74 (0.75-7.00)	3.01 (0.79-7.63)	1.68 (0.46-4.29)	1.60 (0.41-4.22)
Saint Pierre and Miquelon	4	1	0	0.00 (0.00-369.42)	0.00 (0.00-272.81)	0.00 (0.00-600.79)	0.00 (0.00-637.50)
S. Vincent and the Grenadines	15	15	12	9.30 (4.82-16.19)	10.05 (5.18-17.38)	7.35 (3.80-12.84)	8.51 (4.35-14.85)
Saudi Arabia	1	1	0	0.00 (0.00-4.54)	0.00 (0.00-5.76)	0.00 (0.00-0.13)	0.00 (0.00-0.26)
Serbia and Montenegro, Former	3	0	7/-	0.87 (0.35-1.80)	0.86 (0.32-1.99)	-	-
Singapore	3	3	0	0.00 (0.00-4.78)	0.00 (0.00-6.56)	0.00 (0.00-0.23)	0.00 (0.00-0.24)
Slovakia	4	4	3	0.91 (0.19-2.65)	1.23 (0.22-3.70)	0.14 (0.03-0.40)	0.09 (0.02-0.31)
Spain	15	15	494	3.33 (3.05-3.64)	4.35 (3.79-5.00)	0.74 (0.68-0.81)	0.33 (0.30-0.36)
Sri Lanka	1	1	19	2.23 (1.34-3.47)	2.20 (1.30-3.47)	0.97 (0.58-1.51)	1.00 (0.60-1.56)
Suriname	15	15	8	3.12 (1.35-6.13)	3.73 (1.22-8.08)	1.06 (0.46-2.08)	1.31 (0.56-2.58)
Sweden	15	15	31	1.07 (0.73-1.52)	1.48 (0.94-2.23)	0.22 (0.15-0.32)	0.11 (0.07-0.17)
Switzerland	14	14	18	0.88 (0.52-1.39)	1.39 (0.64-2.64)	0.17 (0.10-0.27)	0.08 (0.05-0.14)
Thailand	6	6	1442	31.71 (30.12-33.36)	35.77 (32.76-38.94)	3.71 (3.52-3.90)	3.83 (3.63-4.03)
Trinidad and Tobago	11	11	50	3.31 (2.46-4.36)	3.65 (2.67-4.90)	3.51 (2.60-4.62)	3.83 (2.84-5.04)
Tunisia	2	2	31	8.95 (6.09-12.69)	7.40 (4.46-11.51)	1.44 (0.98-2.04)	1.51 (1.03-2.15)
Turkey	5	5	293	3.61 (3.21-4.05)	3.85 (3.39-4.37)	0.80 (0.71-0.89)	0.94 (0.83-1.06)
Turks and Caicos Islands	11	1	0	0.00 (0.00-84.08)	0.00 (0.00-85.44)	0.00 (0.00-185.28)	0.00 (0.00-279.47)
United Kingdom (UK)	13	13	269	3.20 (2.83-3.61)	4.32 (3.77-4.93)	0.34 (0.30-0.38)	0.23 (0.20-0.26)
UK, England & Wales	14	14	252	3.25 (2.86-3.68)	4.60 (3.99-5.27)	0.33 (0.29-0.37)	0.23 (0.20-0.26)
UK, Northern Ireland	13	13	6	2.42 (0.89-5.25)	3.20 (1.01-7.31)	0.26 (0.10-0.57)	0.20 (0.07-0.45)
UK, Scotland	15	15	34	3.13 (2.17-4.37)	3.57 (2.37-5.13)	0.44 (0.30-0.61)	0.28 (0.19-0.41)
United States of America	15	15	4242	3.88 (3.77-4.00)	3.95 (3.83-4.07)	0.94 (0.91-0.97)	0.64 (0.62-0.66)
Uruguay	14	14	98	9.82 (7.98-11.95)	10.71 (8.48-13.37)	2.09 (1.70-2.54)	1.30 (1.04-1.61)
Venezuela	14	14	227	2.00 (1.74-2.27)	2.02 (1.75-2.31)	0.59 (0.52-0.67)	0.87 (0.76-0.99)
Virgin Islands (USA)	13	13	3	5.89 (1.22-17.13)	5.36 (1.09-16.58)	2.15 (0.44-6.28)	1.55 (0.32-4.98)

Countries are sorted in alphabetical order; sorting by standardised estimates is shown in figure S1 and estimates by year in figure S2.
- denotes no available data
*The number of hypoglycaemia deaths for the two analyses (proportion and rate) was the same, unless indicated (number for proportion/number for rate analysis)

ESM Table 3: Crude and age-standardised proportion differences compared to the overall estimate, by country

Country	Number of hypos	Difference (per 1000 total diabetes deaths) Crude	Standardised
**All fifteen years available**			
Aruba	12	18.98 (5.85; 32.11)	21.93 (8.02; 35.83)
Belize	23	8.88 (3.45; 14.31)	11.38 (5.47; 17.29)
Saint Vincent and the Grenadines	12	4.80 (-0.44; 10.04)	5.55 (0.11; 10.99)
Montserrat	1	2.44 (-11.12; 16.01)	5.53 (-10.74; 21.81)
Chile	461	4.54 (3.72; 5.37)	4.94 (4.10; 5.78)
Bermuda	2	1.13 (-6.66; 8.92)	2.13 (-6.31; 10.57)
Argentina	744	1.36 (0.94; 1.79)	1.66 (1.23; 2.10)
Brazil	3563	0.53 (0.36; 0.71)	0.42 (0.25; 0.59)
Panama	66	0.62 (-0.61; 1.86)	0.39 (-0.82; 1.59)
Peru	168	0.49 (-0.27; 1.24)	0.08 (-0.64; 0.81)
Costa Rica	44	-0.17 (-1.45; 1.11)	-0.05 (-1.34; 1.25)
Spain	494	-1.17 (-1.47; -0.87)	-0.15 (-0.49; 0.19)
Ecuador	224	-0.15 (-0.72; 0.42)	-0.53 (-1.07; 0.02)
United States of America	4242	-0.62 (-0.75; -0.49)	-0.55 (-0.68; -0.42)
Suriname	8	-1.39 (-3.54; 0.77)	-0.76 (-3.12; 1.60)
Mexico	3192	-1.47 (-1.59; -1.35)	-0.81 (-0.94; -0.69)
United Kingdom, Scotland	34	-1.37 (-2.42; -0.32)	-0.93 (-2.05; 0.19)
Puerto Rico	132	-1.36 (-1.90; -0.82)	-1.28 (-1.82; -0.73)
Germany	644	-2.64 (-2.80; -2.49)	-1.62 (-1.80; -1.43)
Norway	25	-2.16 (-3.08; -1.24)	-1.66 (-2.67; -0.65)
Luxembourg	3	-0.97 (-4.96; 3.03)	-1.89 (-5.32; 1.55)
Kuwait	6	-2.37 (-4.08; -0.67)	-2.63 (-4.22; -1.04)
Paraguay	40	-2.78 (-3.32; -2.24)	-2.65 (-3.21; -2.10)
Sweden	31	-3.43 (-3.81; -3.05)	-3.01 (-3.46; -2.57)
Finland	12	-2.93 (-3.82; -2.04)	-3.01 (-3.88; -2.15)
Czech Republic	35	-3.33 (-3.72; -2.94)	-3.28 (-3.68; -2.88)
Poland	77	-3.66 (-3.86; -3.47)	-3.54 (-3.75; -3.33)
Romania	16	-4.01 (-4.26; -3.76)	-3.84 (-4.13; -3.56)
Hungary	17	-4.08 (-4.29; -3.87)	-3.91 (-4.16; -3.67)
Malta	1	-3.87 (-5.11; -2.62)	-4.04 (-5.09; -2.98)
Croatia	2	-4.39 (-4.56; -4.22)	-4.39 (-4.55; -4.22)
**One year unavailable**			
Japan	2868	10.39 (9.85; 10.94)	10.64 (10.09; 11.18)
Guadeloupe	23	8.04 (2.95; 13.14)	9.47 (4.09; 14.84)
Barbados	39	8.53 (4.47; 12.60)	8.36 (4.32; 12.39)
Uruguay	98	5.31 (3.38; 7.25)	6.21 (4.19; 8.23)
Belgium	174	2.98 (1.87; 4.08)	5.67 (4.38; 6.96)
Dominica	5	3.64 (-3.47; 10.75)	5.26 (-2.51; 13.03)
El Salvador	135	3.28 (1.98; 4.59)	3.21 (1.91; 4.51)
Grenada	8	2.38 (-2.37; 7.14)	2.74 (-2.13; 7.61)
Colombia	728	2.82 (2.29; 3.36)	2.73 (2.20; 3.26)
France	588	-0.73 (-1.04; -0.43)	0.47 (0.12; 0.82)
Bahamas	7	0.66 (-3.15; 4.48)	0.44 (-3.29; 4.17)
UK, England & Wales	252	-1.25 (-1.65; -0.84)	0.11 (-0.37; 0.59)
Nicaragua	58	-1.13 (-2.00; -0.26)	-0.33 (-1.29; 0.63)
Israel	116	-1.08 (-1.70; -0.46)	-0.62 (-1.29; 0.04)
Australia	153	-1.64 (-2.10; -1.18)	-1.16 (-1.65; -0.66)
Saint Lucia	4	-1.76 (-4.45; 0.92)	-1.48 (-4.30; 1.33)
Netherlands	125	-1.88 (-2.34; -1.42)	-1.61 (-2.09; -1.12)
Cuba	79	-1.81 (-2.40; -1.21)	-1.72 (-2.33; -1.12)
Venezuela	227	-2.51 (-2.77; -2.24)	-2.48 (-2.74; -2.21)
Switzerland	18	-3.62 (-4.03; -3.21)	-3.11 (-3.62; -2.59)
Lithuania	7	-2.82 (-4.07; -1.58)	-3.29 (-4.34; -2.23)
Kyrgyzstan	0	-4.50 (-4.56; -4.45)	-4.49 (-4.55; -4.44)
**Two years unavailable**			
French Guiana	5	11.57 (-2.40; 25.55)	7.72 (-4.49; 19.93)
Saint Kitts and Nevis	4	7.66 (-4.19; 19.50)	6.99 (-4.53; 18.50)
Martinique	11	2.20 (-1.75; 6.15)	4.33 (-0.20; 8.85)
Reunion	24	3.32 (0.20; 6.43)	4.26 (0.97; 7.56)
Dominican Republic	119	3.33 (1.93; 4.73)	3.06 (1.68; 4.44)
Virgin Islands (USA)	3	1.39 (-5.26; 8.04)	0.87 (-5.48; 7.21)
Hong Kong SAR	37	0.64 (-1.01; 2.29)	0.43 (-1.19; 2.04)
United Kingdom	269	-1.30 (-1.69; -0.92)	-0.17 (-0.62; 0.27)
UK, Northern Ireland	6	-2.09 (-4.02; -0.16)	-1.29 (-3.52; 0.93)
Antigua and Barbuda	2	-1.18 (-5.78; 3.42)	-1.33 (-5.82; 3.15)
Canada	266	-1.73 (-2.07; -1.39)	-1.36 (-1.71; -1.00)
Denmark	25	-3.07 (-3.63; -2.50)	-3.02 (-3.59; -2.45)
New Zealand	5	-4.04 (-4.45; -3.63)	-3.93 (-4.38; -3.48)
Austria	5	-4.38 (-4.50; -4.26)	-4.22 (-4.39; -4.05)
Anguilla	0	-4.50 (-4.56; -4.45)	-4.49 (-4.55; -4.44)
**Three or more years unavailable**			
Kiribati	8	120.50 (39.47; 201.52)	278.64 (168.26; 389.02)
Thailand	1442	27.21 (25.60; 28.82)	31.28 (29.57; 32.98)
Oman	7	32.15 (5.50; 58.79)	27.18 (2.34; 52.01)
Maldives	3	28.46 (-8.22; 65.15)	23.51 (-10.39; 57.41)
Philippines	2503	11.06 (10.45; 11.66)	21.47 (20.69; 22.25)
Guyana	60	7.79 (4.70; 10.88)	8.98 (5.74; 12.21)
Bolivia	7	5.54 (-1.86; 12.94)	6.63 (-1.16; 14.41)
Iceland	1	-0.21 (-8.61; 8.18)	5.01 (-7.45; 17.47)
Honduras	14	1.16 (-1.80; 4.12)	3.70 (0.15; 7.26)
Tunisia	31	4.45 (1.31; 7.59)	2.91 (0.05; 5.76)
Guatemala	327	3.17 (2.34; 4.00)	2.75 (1.94; 3.56)
Ireland	18	0.57 (-1.77; 2.91)	2.44 (-0.29; 5.17)
Morocco	105	4.26 (2.59; 5.93)	2.37 (0.89; 3.85)
Haiti	4	2.42 (-4.34; 9.18)	1.98 (-4.56; 8.51)
Fiji	54	0.07 (-1.15; 1.28)	1.62 (0.21; 3.03)
Jamaica	77	-0.04 (-1.04; 0.95)	-0.02 (-1.02; 0.97)
Georgia	21	-1.72 (-2.91; -0.53)	-0.41 (-1.85; 1.04)
Turkey	293	-0.89 (-1.31; -0.47)	-0.64 (-1.07; -0.21)
Trinidad and Tobago	50	-1.19 (-2.11; -0.27)	-0.84 (-1.81; 0.12)
Mauritius	56	-1.86 (-2.56; -1.17)	-1.82 (-2.51; -1.12)
Rodrigues	1	-2.15 (-6.76; 2.46)	-2.23 (-6.75; 2.28)
Sri Lanka	19	-2.28 (-3.28; -1.28)	-2.30 (-3.29; -1.30)
Bulgaria	3	-2.35 (-4.78; 0.08)	-2.77 (-4.95; -0.60)
Jordan	6	-3.18 (-4.24; -2.11)	-3.07 (-4.17; -1.97)
Portugal	66	-3.05 (-3.40; -2.69)	-3.08 (-3.43; -2.73)
Cyprus	6	-2.76 (-4.15; -1.36)	-3.09 (-4.34; -1.83)
Italy	213	-3.20 (-3.38; -3.02)	-3.13 (-3.32; -2.94)
Slovakia	3	-3.60 (-4.62; -2.57)	-3.27 (-4.46; -2.08)
Serbia and Montenegro, Former	7	-3.63 (-4.28; -2.98)	-3.64 (-4.28; -2.99)
Latvia	2	-3.91 (-4.73; -3.08)	-3.71 (-4.66; -2.76)
Armenia	0	-4.50 (-4.56; -4.45)	-4.49 (-4.55; -4.44)
British Virgin Islands	0	-4.50 (-4.56; -4.45)	-4.49 (-4.55; -4.44)
Cayman Islands	0	-4.50 (-4.56; -4.45)	-4.49 (-4.55; -4.44)
Estonia	0	-4.50 (-4.56; -4.45)	-4.49 (-4.55; -4.44)
Mayotte	0	-4.50 (-4.56; -4.45)	-4.49 (-4.55; -4.44)
Netherlands Antilles	0	-4.50 (-4.56; -4.45)	-4.49 (-4.55; -4.44)
Occupied Palestinian Territory	0	-4.50 (-4.56; -4.45)	-4.49 (-4.55; -4.44)
Republic of Moldova	0	-4.50 (-4.56; -4.45)	-4.49 (-4.55; -4.44)
Saint Pierre and Miquelon	0	-4.50 (-4.56; -4.45)	-4.49 (-4.55; -4.44)
Saudi Arabia	0	-4.50 (-4.56; -4.45)	-4.49 (-4.55; -4.44)
Singapore	0	-4.50 (-4.56; -4.45)	-4.49 (-4.55; -4.44)
Turks and Caicos Islands	0	-4.50 (-4.56; -4.45)	-4.49 (-4.55; -4.44)

Differences are reported with 95% CI. Within each group of available years, countries are sorted by standardised difference.

ESM Table 4: Crude and age-standardised odds ratios compared to the overall estimate, for countries with three or more years unavailable

Country	Number of hypos	Proportion (per 1000 total diabetes deaths)		Odds ratio	
		Crude	Standardised	Crude	Standardised
Kiribati	8	125.00	283.13	27.76 (13.88-55.51)	62.93 (39.69-99.77)
Thailand	1442	31.71	35.77	7.04 (6.68-7.43)	7.96 (7.57-8.37)
Oman	7	36.65	31.67	8.14 (3.88-17.07)	6.99 (3.14-15.56)
Maldives	3	32.97	28.00	7.32 (2.36-22.70)	6.11 (1.77-21.12)
Philippines	2503	15.56	25.96	3.46 (3.32-3.60)	5.78 (5.59-5.97)
Guyana	60	12.29	13.47	2.73 (2.12-3.52)	3.00 (2.36-3.82)
Bolivia	7	10.04	11.12	2.23 (1.06-4.68)	2.49 (1.23-5.02)
Iceland	1	4.29	9.51	0.95 (0.13-6.77)	2.10 (0.56-7.88)
Honduras	14	5.66	8.20	1.26 (0.74-2.12)	1.83 (1.18-2.82)
Tunisia	31	8.95	7.40	1.99 (1.40-2.83)	1.65 (1.12-2.42)
Guatemala	327	7.68	7.25	1.70 (1.53-1.90)	1.61 (1.44-1.80)
Ireland	18	5.07	6.93	1.13 (0.71-1.79)	1.54 (1.04-2.29)
Morocco	105	8.76	6.86	1.95 (1.61-2.36)	1.53 (1.23-1.90)
Haiti	4	6.92	6.47	1.54 (0.58-4.10)	1.42 (0.51-3.95)
Fiji	54	4.57	6.11	1.01 (0.78-1.33)	1.36 (1.08-1.71)
Jamaica	77	4.46	4.47	0.99 (0.79-1.24)	1.00 (0.80-1.24)
Georgia	21	2.78	4.09	0.62 (0.40-0.95)	0.91 (0.64-1.30)
Turkey	293	3.61	3.85	0.80 (0.71-0.90)	0.86 (0.77-0.96)
Trinidad and Tobago	50	3.31	3.65	0.74 (0.56-0.97)	0.81 (0.62-1.06)
Mauritius	56	2.64	2.68	0.59 (0.45-0.76)	0.60 (0.46-0.77)
Rodrigues	1	2.35	2.26	0.52 (0.07-3.71)	0.52 (0.07-3.72)
Sri Lanka	19	2.23	2.20	0.49 (0.32-0.78)	0.49 (0.31-0.77)
Bulgaria	3	2.15	1.72	0.48 (0.15-1.48)	0.38 (0.11-1.36)
Jordan	6	1.33	1.43	0.29 (0.13-0.66)	0.32 (0.15-0.68)
Portugal	66	1.46	1.41	0.32 (0.25-0.41)	0.31 (0.25-0.40)
Cyprus	6	1.75	1.41	0.39 (0.17-0.86)	0.31 (0.13-0.76)
Italy	213	1.30	1.36	0.29 (0.25-0.33)	0.30 (0.27-0.35)
Slovakia	3	0.91	1.22	0.20 (0.06-0.62)	0.28 (0.10-0.73)
Serbia and Montenegro, Former	7	0.87	0.86	0.19 (0.09-0.41)	0.19 (0.09-0.40)
Latvia	2	0.59	0.78	0.13 (0.03-0.53)	0.17 (0.05-0.58)
Armenia	0	0	0	-	-
British Virgin Islands	0	0	0	-	-
Cayman Islands	0	0	0	-	-
Estonia	0	0	0	-	-
Mayotte	0	0	0	-	-
Netherlands Antilles	0	0	0	-	-
Occupied Palestinian Territory	0	0	0	-	-
Republic of Moldova	0	0	0	-	-
Saint Pierre and Miquelon	0	0	0	-	-
Saudi Arabia	0	0	0	-	-
Singapore	0	0	0	-	-
Turks and Caicos Islands	0	0	0	-	-

Odds ratios are reported with 95% CI. Countries are sorted by standardised odds ratio.

ESM Table 5: Crude and age-standardised rate differences compared to the overall estimate, by country

Country	Number of hypos	Difference (per 1 million person-years) Crude	Standardised
**All fifteen years available**			
Belize	23	4.05 (1.95; 6.14)	10.55 (7.44; 13.67)
Saint Vincent and the Grenadines	12	6.27 (2.11; 10.43)	7.72 (3.25; 12.19)
Aruba	12	6.97 (2.41; 11.52)	6.65 (2.27; 11.02)
Mexico	3192	0.79 (0.73; 0.86)	1.69 (1.62; 1.77)
Chile	461	0.79 (0.62; 0.96)	0.79 (0.64; 0.95)
Puerto Rico	132	1.27 (0.87; 1.67)	0.79 (0.46; 1.11)
Brazil	3563	0.16 (0.12; 0.20)	0.76 (0.71; 0.80)
Panama	66	0.20 (-0.11; 0.51)	0.67 (0.34; 1.00)
Bermuda	2	1.03 (-1.89; 3.95)	0.59 (-1.77; 2.95)
Suriname	8	-0.02 (-0.76; 0.71)	0.53 (-0.29; 1.35)
Ecuador	224	-0.03 (-0.17; 0.11)	0.50 (0.35; 0.65)
Argentina	744	0.16 (0.07; 0.25)	0.21 (0.13; 0.29)
Costa Rica	44	-0.41 (-0.61; -0.21)	-0.04 (-0.25; 0.17)
Paraguay	40	-0.63 (-0.77; -0.49)	-0.10 (-0.27; 0.07)
United States of America	4242	-0.14 (-0.17; -0.11)	-0.14 (-0.17; -0.12)
Kuwait	6	-0.93 (-1.05; -0.81)	-0.20 (-0.44; 0.04)
Peru	168	-0.68 (-0.75; -0.62)	-0.28 (-0.35; -0.21)
Spain	494	-0.34 (-0.41; -0.27)	-0.46 (-0.50; -0.41)
United Kingdom, Scotland	34	-0.64 (-0.79; -0.49)	-0.50 (-0.62; -0.39)
Germany	644	-0.55 (-0.59; -0.51)	-0.51 (-0.54; -0.48)
Luxembourg	3	-0.67 (-1.13; -0.20)	-0.60 (-0.91; -0.28)
Norway	25	-0.73 (-0.87; -0.59)	-0.60 (-0.70; -0.50)
Finland	12	-0.93 (-1.01; -0.84)	-0.64 (-0.73; -0.56)
Czech Republic	35	-0.85 (-0.93; -0.78)	-0.66 (-0.72; -0.60)
Sweden	31	-0.85 (-0.93; -0.77)	-0.67 (-0.73; -0.62)
Malta	1	-0.91 (-1.24; -0.59)	-0.69 (-0.94; -0.45)
Poland	77	-0.95 (-0.98; -0.91)	-0.69 (-0.72; -0.66)
Hungary	17	-0.97 (-1.02; -0.91)	-0.71 (-0.76; -0.67)
Romania	16	-1.03 (-1.06; -1.00)	-0.75 (-0.77; -0.72)
Croatia	2	-1.05 (-1.09; -1.00)	-0.77 (-0.80; -0.74)
**One year unavailable**			
Barbados	39	9.02 (5.85; 12.19)	5.40 (2.92; 7.88)
Grenada	8	4.42 (0.61; 8.23)	5.22 (1.23; 9.20)
Guadeloupe	23	2.57 (1.08; 4.06)	1.69 (0.46; 2.92)
El Salvador	135	0.54 (0.26; 0.81)	1.21 (0.91; 1.52)
Bahamas	7	0.40 (-0.70; 1.49)	0.96 (-0.23; 2.15)
Colombia	728	0.10 (0.02; 0.19)	0.84 (0.74; 0.95)
Saint Lucia	4	0.60 (-1.05; 2.24)	0.81 (-0.79; 2.41)
Uruguay	98	1.01 (0.60; 1.42)	0.51 (0.19; 0.84)
Nicaragua	58	-0.32 (-0.52; -0.13)	0.50 (0.25; 0.76)
Venezuela	227	-0.49 (-0.57; -0.41)	0.08 (-0.01; 0.18)
Israel	116	0.13 (-0.09; 0.34)	0.07 (-0.12; 0.25)
Japan	2868	0.54 (0.48; 0.60)	-0.11 (-0.15; -0.07)
Belgium	174	0.08 (-0.09; 0.26)	-0.17 (-0.29; -0.04)
Cuba	79	-0.58 (-0.69; -0.47)	-0.40 (-0.50; -0.30)
France	588	-0.40 (-0.46; -0.34)	-0.44 (-0.48; -0.40)
Australia	153	-0.56 (-0.65; -0.48)	-0.46 (-0.52; -0.39)
Netherlands	125	-0.53 (-0.63; -0.44)	-0.49 (-0.56; -0.42)
UK, England & Wales	252	-0.75 (-0.79; -0.71)	-0.56 (-0.59; -0.52)
Lithuania	7	-0.92 (-1.04; -0.81)	-0.65 (-0.76; -0.55)
Switzerland	18	-0.91 (-0.99; -0.83)	-0.70 (-0.76; -0.65)
Kyrgyzstan	0	-1.08 (-1.09; -1.07)	-0.79 (-0.80; -0.77)
**Two years unavailable**			
French Guiana	5	0.71 (-0.86; 2.29)	2.03 (0.06; 4.00)
Reunion	24	1.21 (0.30; 2.13)	1.50 (0.59; 2.42)
Antigua and Barbuda	2	0.75 (-1.79; 3.29)	0.97 (-1.51; 3.46)
Virgin Islands (USA)	3	1.07 (-1.36; 3.50)	0.77 (-1.30; 2.83)
Martinique	11	1.07 (-0.20; 2.33)	0.49 (-0.49; 1.47)
Dominican Republic	119	-0.10 (-0.28; 0.07)	0.49 (0.29; 0.69)
Canada	266	-0.45 (-0.53; -0.38)	-0.40 (-0.46; -0.34)

Country	Number of hypos	Difference (per 1 million person-years) Crude	Standardised
Hong Kong SAR	37	-0.67 (-0.80; -0.54)	-0.55 (-0.65; -0.44)
United Kingdom	269	-0.74 (-0.78; -0.70)	-0.55 (-0.59; -0.52)
UK, Northern Ireland	6	-0.82 (-1.03; -0.61)	-0.59 (-0.77; -0.41)
Denmark	25	-0.73 (-0.87; -0.59)	-0.60 (-0.70; -0.50)
New Zealand	5	-0.99 (-1.07; -0.90)	-0.71 (-0.78; -0.64)
Austria	5	-1.03 (-1.08; -0.99)	-0.76 (-0.79; -0.72)
**Three or more years unavailable**			
Kiribati	8	45.91 (13.35; 78.47)	109.12 (59.32; 158.92)
Guyana	60	5.61 (3.92; 7.30)	10.39 (8.20; 12.57)
Dominica	2	13.22 (-6.60; 33.04)	10.37 (-7.13; 27.88)
Fiji	54	4.80 (3.23; 6.37)	9.56 (7.48; 11.64)
Philippines	2503	2.14 (2.01; 2.27)	7.96 (7.76; 8.17)
Oman	7	1.30 (-0.46; 3.06)	4.49 (1.87; 7.11)
Mauritius	56	3.42 (2.24; 4.59)	3.60 (2.43; 4.76)
Thailand	1442	2.63 (2.44; 2.82)	3.04 (2.85; 3.24)
Trinidad and Tobago	50	2.43 (1.45; 3.40)	3.04 (2.03; 4.06)
Maldives	3	1.21 (-1.38; 3.81)	3.00 (-0.33; 6.33)
Guatemala	327	1.16 (0.92; 1.41)	2.82 (2.51; 3.12)
Jamaica	77	1.80 (1.16; 2.44)	2.02 (1.38; 2.65)
Tunisia	31	0.36 (-0.15; 0.87)	0.73 (0.21; 1.25)
Sri Lanka	19	-0.11 (-0.55; 0.32)	0.21 (-0.23; 0.65)
Turkey	293	-0.28 (-0.38; -0.19)	0.16 (0.06; 0.26)
Morocco	105	-0.43 (-0.55; -0.30)	-0.01 (-0.15; 0.12)
Jordan	6	-0.84 (-1.03; -0.66)	-0.30 (-0.57; -0.03)
Ireland	18	-0.52 (-0.78; -0.26)	-0.34 (-0.57; -0.11)
Honduras	14	-0.77 (-0.93; -0.61)	-0.40 (-0.58; -0.22)
Cyprus	6	-0.52 (-0.97; -0.08)	-0.42 (-0.78; -0.06)
Georgia	21	-0.59 (-0.80; -0.38)	-0.45 (-0.62; -0.28)
Bolivia	7	-0.87 (-1.03; -0.72)	-0.47 (-0.66; -0.28)
Iceland	1	-0.74 (-1.40; -0.08)	-0.47 (-1.11; 0.16)
Portugal	66	-0.45 (-0.60; -0.30)	-0.53 (-0.63; -0.44)
Haiti	4	-0.97 (-1.08; -0.86)	-0.59 (-0.74; -0.45)
Bulgaria	3	-0.67 (-1.13; -0.20)	-0.60 (-0.91; -0.29)
Italy	213	-0.63 (-0.69; -0.57)	-0.63 (-0.67; -0.59)
Latvia	2	-0.94 (-1.13; -0.75)	-0.70 (-0.85; -0.55)
Slovakia	3	-0.94 (-1.10; -0.78)	-0.70 (-0.82; -0.57)
Armenia	0	-1.08 (-1.09; -1.07)	-0.79 (-0.80; -0.77)
British Virgin Islands	0	-1.08 (-1.09; -1.07)	-0.79 (-0.80; -0.77)
Cayman Islands	0	-1.08 (-1.09; -1.07)	-0.79 (-0.80; -0.77)
Estonia	0	-1.08 (-1.09; -1.07)	-0.79 (-0.80; -0.77)
Mayotte	0	-1.08 (-1.09; -1.07)	-0.79 (-0.80; -0.77)
Montserrat	0	-1.08 (-1.09; -1.07)	-0.79 (-0.80; -0.77)
Occupied Palestinian Territory	0	-1.08 (-1.09; -1.07)	-0.79 (-0.80; -0.77)
Republic of Moldova	0	-1.08 (-1.09; -1.07)	-0.79 (-0.80; -0.77)
Saint Kitts and Nevis	0	-1.08 (-1.09; -1.07)	-0.79 (-0.80; -0.77)
Saint Pierre and Miquelon	0	-1.08 (-1.09; -1.07)	-0.79 (-0.80; -0.77)
Saudi Arabia	0	-1.08 (-1.09; -1.07)	-0.79 (-0.80; -0.77)
Singapore	0	-1.08 (-1.09; -1.07)	-0.79 (-0.80; -0.77)
Turks and Caicos Islands	0	-1.08 (-1.09; -1.07)	-0.79 (-0.80; -0.77)

Differences are reported with 95% CI. Within each groups of available years, countries are sorted by standardised difference.

ESM Table 6: Crude and age-standardised rate ratios compared to the overall estimate, for countries with three or more years unavailable

Country	Number of hypos	Rate (per 1 million person-years) Crude	Standardised	Rate Ratio Crude	Standardised
Kiribati	8	46.99	109.90	43.54 (21.77-87.08)	139.86 (88.87-220.11)
Dominica	2	14.30	11.16	13.25 (3.31-52.99)	14.57 (3.09-68.61)
Guyana	60	6.69	11.17	6.20 (4.81-7.99)	14.23 (11.69-17.31)
Fiji	54	5.88	10.35	5.45 (4.17-7.12)	13.18 (10.78-16.13)
Philippines	2503	3.22	8.75	2.98 (2.86-3.11)	11.14 (10.84-11.45)
Oman	7	2.38	5.28	2.20 (1.05-4.62)	6.71 (4.07-11.03)
Mauritius	56	4.50	4.38	4.17 (3.21-5.42)	5.58 (4.28-7.28)
Thailand	1442	3.71	3.83	3.43 (3.26-3.62)	4.88 (4.63-5.14)
Trinidad and Tobago	50	3.50	3.83	3.25 (2.46-4.29)	4.87 (3.74-6.36)
Maldives	3	2.29	3.78	2.13 (0.69-6.59)	4.77 (1.97-11.57)
Guatemala	327	2.24	3.60	2.08 (1.86-2.32)	4.58 (4.20-5.00)
Jamaica	77	2.88	2.80	2.67 (2.13-3.34)	3.57 (2.84-4.48)
Tunisia	31	1.44	1.51	1.33 (0.94-1.90)	1.93 (1.37-2.72)
Sri Lanka	19	0.97	1.00	0.90 (0.57-1.40)	1.27 (0.81-1.98)
Turkey	293	0.80	0.94	0.74 (0.66-0.83)	1.20 (1.08-1.33)
Morocco	105	0.65	0.77	0.61 (0.50-0.73)	0.98 (0.82-1.17)
Jordan	6	0.23	0.48	0.22 (0.10-0.48)	0.62 (0.35-1.08)
Ireland	18	0.56	0.44	0.52 (0.33-0.83)	0.56 (0.34-0.95)
Honduras	14	0.31	0.39	0.29 (0.17-0.48)	0.49 (0.31-0.79)
Cyprus	6	0.55	0.36	0.51 (0.23-1.14)	0.46 (0.17-1.24)
Georgia	21	0.49	0.33	0.45 (0.30-0.70)	0.43 (0.25-0.71)
Bolivia	7	0.20	0.32	0.19 (0.09-0.40)	0.41 (0.23-0.74)
Iceland	1	0.34	0.31	0.31 (0.04-2.22)	0.39 (0.05-3.05)
Portugal	66	0.63	0.25	0.58 (0.46-0.74)	0.32 (0.22-0.47)
Haiti	4	0.11	0.19	0.10 (0.04-0.28)	0.25 (0.12-0.52)
Bulgaria	3	0.41	0.18	0.38 (0.12-1.19)	0.23 (0.04-1.27)
Italy	213	0.45	0.15	0.42 (0.36-0.48)	0.20 (0.16-0.25)
Slovakia	3	0.14	0.09	0.13 (0.04-0.40)	0.12 (0.03-0.47)
Latvia	2	0.14	0.09	0.13 (0.03-0.51)	0.11 (0.02-0.64)
Armenia	0	0	0	-	-
British Virgin Islands	0	0	0	-	-
Cayman Islands	0	0	0	-	-
Estonia	0	0	0	-	-
Mayotte	0	0	0	-	-
Montserrat	0	0	0	-	-
Occupied Palestinian Territory	0	0	0	-	-
Republic of Moldova	0	0	0	-	-
Saint Kitts and Nevis	0	0	0	-	-
Saint Pierre and Miquelon	0	0	0	-	-
Saudi Arabia	0	0	0	-	-
Singapore	0	0	0	-	-
Turks and Caicos Islands	0	0	0	-	-

Rate ratios are reported with 95% CI. Countries are sorted by standardised rate ratio.

ESM Table 7: Age–standardised proportion and rate differences compared to their respective overall estimates, by geographical area and socio–demographic development

Country	SDI	WBI	Geographical Area	Proportion	Rate
Mauritius	High-middle	Upper-middle	Africa	10	10
Tunisia	Middle	Lower-middle	Africa	2	2
Morocco	Low-middle	Lower-middle	Africa	5	5
Mayotte	-	-	Africa	2	2
Reunion	-	-	Africa	13	13
Rodrigues	-	-	Africa	10	0
Hong Kong SAR	High	High	Asia	13	13
Japan	High	High	Asia	14	14
Kuwait	High	High	Asia	15	15
Singapore	High	High	Asia	3	3
Armenia	High-middle	Lower-middle	Asia	1	1
Georgia	High-middle	Upper-middle	Asia	10	10
Oman	High-middle	High	Asia	1	1
Sri Lanka	High-middle	Lower-middle	Asia	1	1
Thailand	High-middle	Upper-middle	Asia	6	6
Kyrgyzstan	Middle	-	Asia	14	14
Maldives	Middle	Upper-middle	Asia	4	4
Philippines	Middle	Lower-middle	Asia	9	9
Saudi Arabia	-	High	Asia	1	1
Antigua and Barbuda	High	High	Caribbean	13	13
Bahamas	High	High	Caribbean	14	14
Bermuda	High	High	Caribbean	15	15
Puerto Rico	High	High	Caribbean	15	15
Trinidad and Tobago	High	High	Caribbean	11	11
Virgin Islands (USA)	High	High	Caribbean	13	13
Barbados	High-middle	High	Caribbean	14	14
Cuba	High-middle	Upper-middle	Caribbean	14	14
Dominica	High-middle	Upper-middle	Caribbean	14	2
Dominican Republic	High-middle	Upper-middle	Caribbean	13	13
Grenada	High-middle	Upper-middle	Caribbean	14	14
Jamaica	High-middle	Upper-middle	Caribbean	10	10
Saint Lucia	High-middle	Upper-middle	Caribbean	14	14
Saint Vincent and the Grenadines	High-middle	Upper-middle	Caribbean	15	15
Belize	Middle	Upper-middle	Caribbean	15	15
Haiti	Low-middle	Low	Caribbean	4	4
Anguilla	-	-	Caribbean	13	0
Aruba	-	High	Caribbean	15	15
British Virgin Islands	-	High	Caribbean	9	2
Cayman Islands	-	High	Caribbean	12	5
Guadeloupe	-	-	Caribbean	14	14
Martinique	-	-	Caribbean	13	13
Montserrat	-	-	Caribbean	15	3
Netherlands Antilles	-	-	Caribbean	1	0
Saint Kitts and Nevis	-	High	Caribbean	13	1
Turks and Caicos Islands	-	High	Caribbean	11	1
Costa Rica	High-middle	Upper-middle	Central America	15	15
Panama	High-middle	Upper-middle	Central America	15	15
El Salvador	Middle	Lower-middle	Central America	14	14
Honduras	Middle	Lower-middle	Central America	6	6
Nicaragua	Middle	Lower-middle	Central America	14	14
Guatemala	Low-middle	Lower-middle	Central America	10	10
Mexico	-	Upper-middle	Central America	15	15
Austria	High	High	Europe	13	13
Belgium	High	High	Europe	14	14
Cyprus	High	High	Europe	10	10
Czech Republic	High	High	Europe	15	15
Denmark	High	High	Europe	13	13

Country	SDI	WBI	Geographical Area	Proportion	Rate
Estonia	High	High	Europe	5	5
Finland	High	High	Europe	15	15
France	High	High	Europe	14	14
Germany	High	High	Europe	15	15
Hungary	High	High	Europe	15	15
Iceland	High	High	Europe	10	10
Ireland	High	High	Europe	7	7
Italy	High	High	Europe	8	8
Latvia	High	High	Europe	7	7
Lithuania	High	High	Europe	14	14
Luxembourg	High	High	Europe	15	15
Netherlands	High	High	Europe	14	14
Norway	High	High	Europe	15	15
Poland	High	High	Europe	15	15
Slovakia	High	High	Europe	4	4
Sweden	High	High	Europe	15	15
Switzerland	High	High	Europe	14	14
United Kingdom (UK)	High	High	Europe	13	13
UK, England & Wales	High	-	Europe	14	14
UK, Scotland	High	-	Europe	15	15
Bulgaria	High-middle	Upper-middle	Europe	1	1
Croatia	High-middle	High	Europe	15	15
Malta	High-middle	High	Europe	15	15
Portugal	High-middle	High	Europe	10	10
Republic of Moldova	High-middle	Lower-middle	Europe	8	8
Romania	High-middle	Upper-middle	Europe	15	15
Serbia and Montenegro, Former	High-middle	Upper-middle	Europe	3	0
Spain	High-middle	High	Europe	15	15
Turkey	High-middle	Upper-middle	Europe	5	5
UK, Northern Ireland	High-middle	-	Europe	13	13
Israel	High	High	Middle East	14	14
Jordan	High-middle	Upper-middle	Middle East	4	4
Occupied Palestinian Territory	Middle	Lower-middle	Middle East	2	2
Canada	High	High	North America	13	13
United States of America	High	High	North America	15	15
Saint Pierre and Miquelon	-	-	North America	4	1
Australia	High	High	Oceania	14	14
New Zealand	High	High	Oceania	13	13
Fiji	High-middle	Upper-middle	Oceania	11	11
Kiribati	Low-middle	Lower-middle	Oceania	2	2
Argentina	High-middle	Upper-middle	South America	15	15
Chile	High-middle	High	South America	15	15
Colombia	High-middle	Upper-middle	South America	14	14
Ecuador	High-middle	Upper-middle	South America	15	15
Peru	High-middle	Upper-middle	South America	15	15
Suriname	High-middle	Upper-middle	South America	15	15
Uruguay	High-middle	High	South America	14	14
Venezuela	High-middle	Upper-middle	South America	14	14
Bolivia	Middle	Lower-middle	South America	4	4
Guyana	Middle	Upper-middle	South America	12	12
Paraguay	Middle	Upper-middle	South America	15	15
Brazil	-	Upper-middle	South America	15	15
French Guiana	-	Upper-middle	South America	13	13

Countries are sorted by geographical area and socio–demographic development. Numbers indicate years available and white cells denote no available data or no hypoglycaemia deaths

- indicates no data available for socio-demographic development

SDI: Socio-demographic index (2015); WBI: World Bank income index (2017)

Statistically significant higher

Not statistically significant higher

Not statistically significant lower

Statistically significant lower

ESM Figure 1: Flow-diagram of data search process

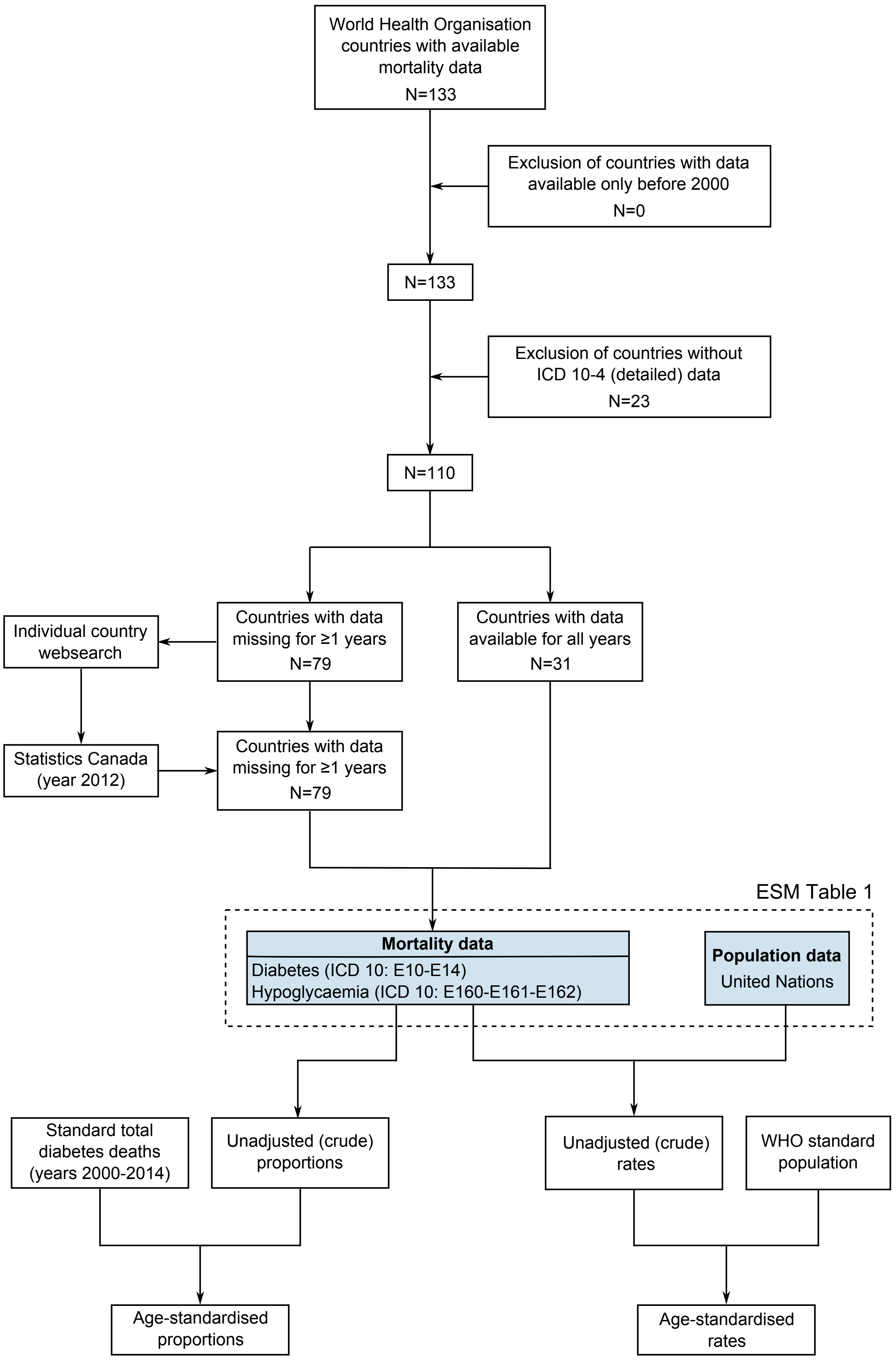

ESM Figure 2: Country–specific crude and age–standardised proportion and rate

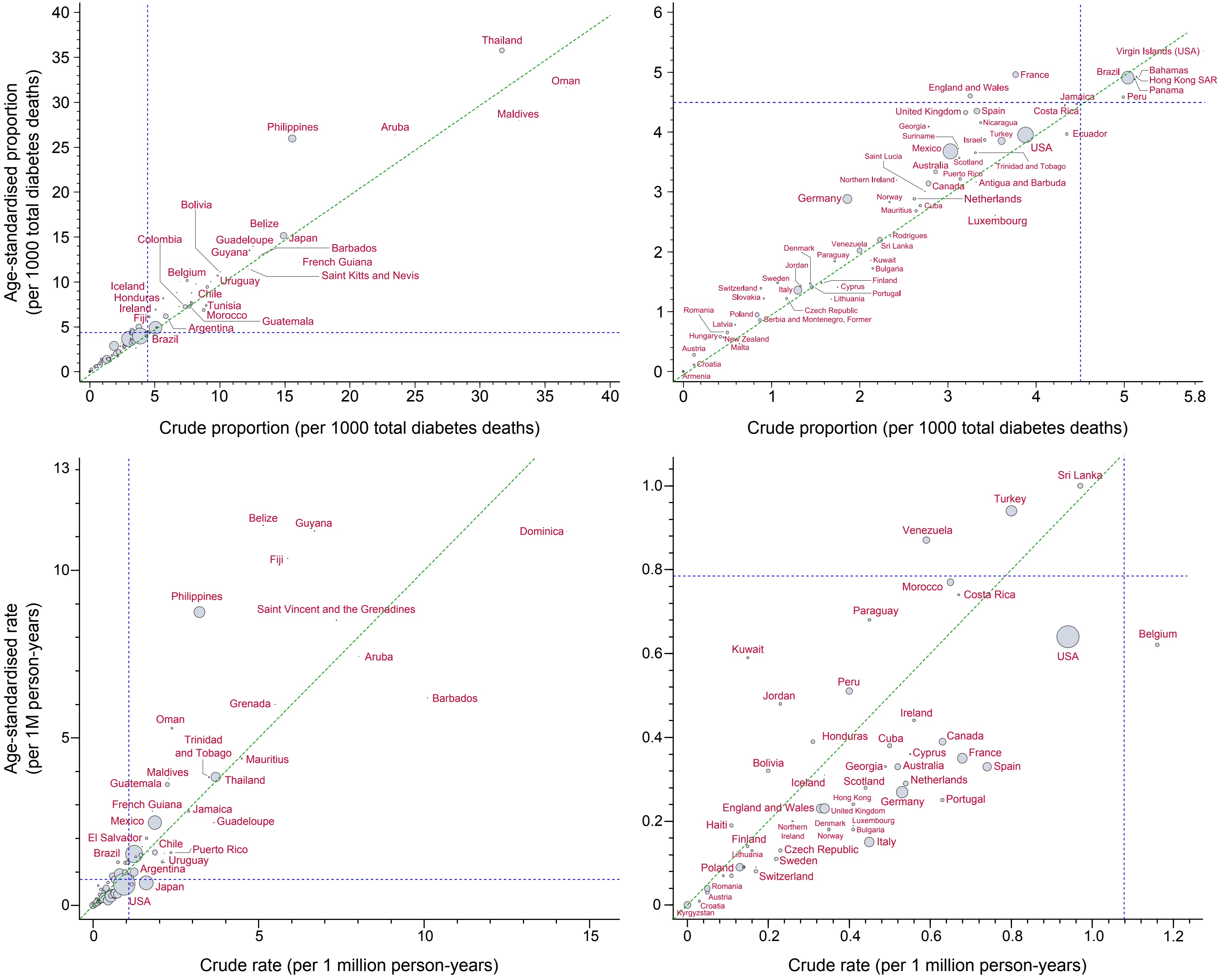

The size of the circles is proportional to mean total hypoglycaemia-related deaths (for proportions) and mean person–years (for rates) during the years of observations. Dotted vertical and horizontal lines indicate the mean overall estimate for all countries and dotted diagonal lines represent lines of equality; estimates with 95% confidence intervals are reported in ESM Table 2. Countries with estimates lower than the overall means are magnified in the right graphs. Kiribati is not shown.

ESM Figure 3: Crude and age-standardised proportions and rates, by country

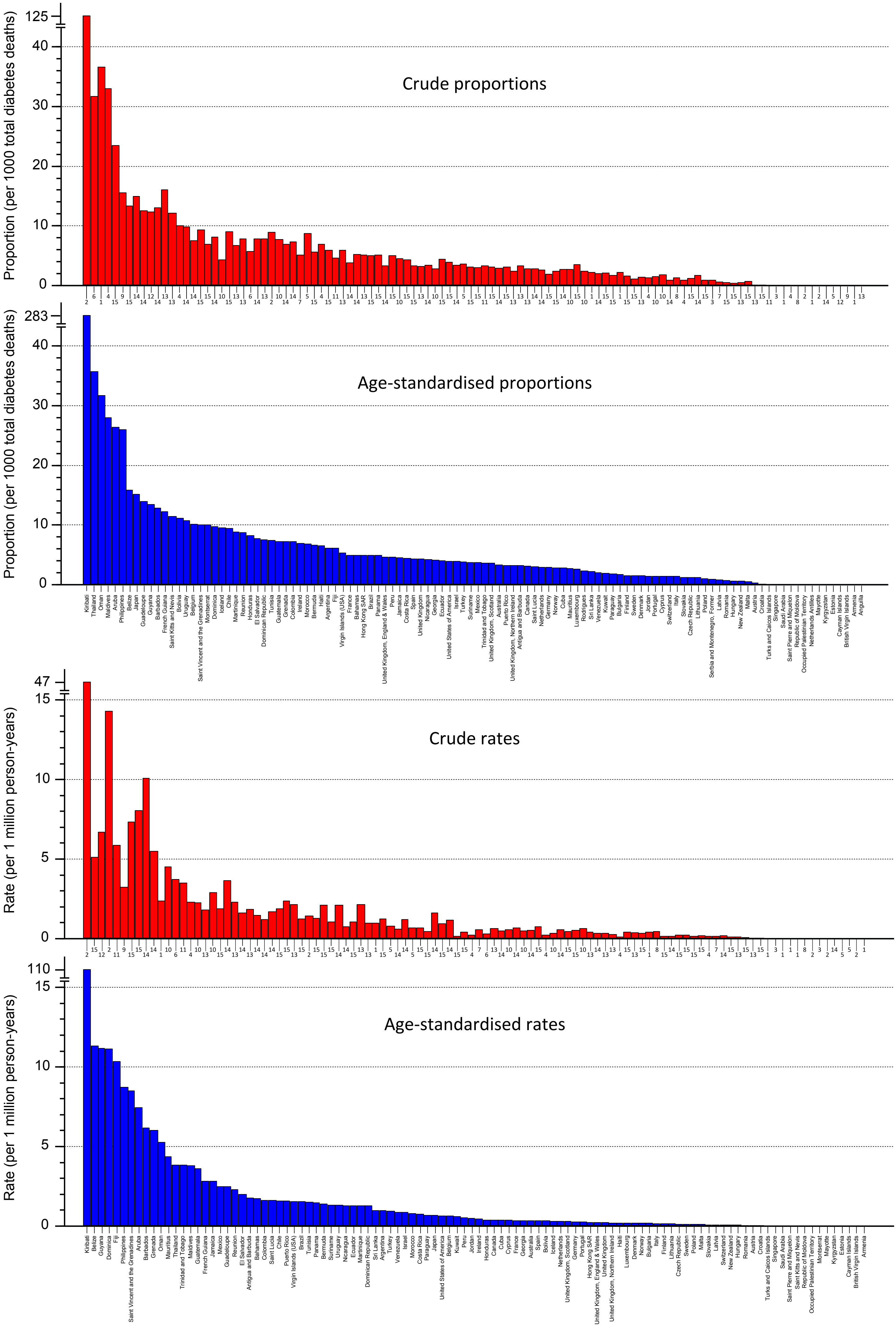

Countries are sorted by decreasing age-standardised estimates of hypoglycaemia-related deaths. Numbers below the x-axes indicate country-specific available years

ESM Figure 4: Year-specific crude and age-standardised proportions and rates, by country

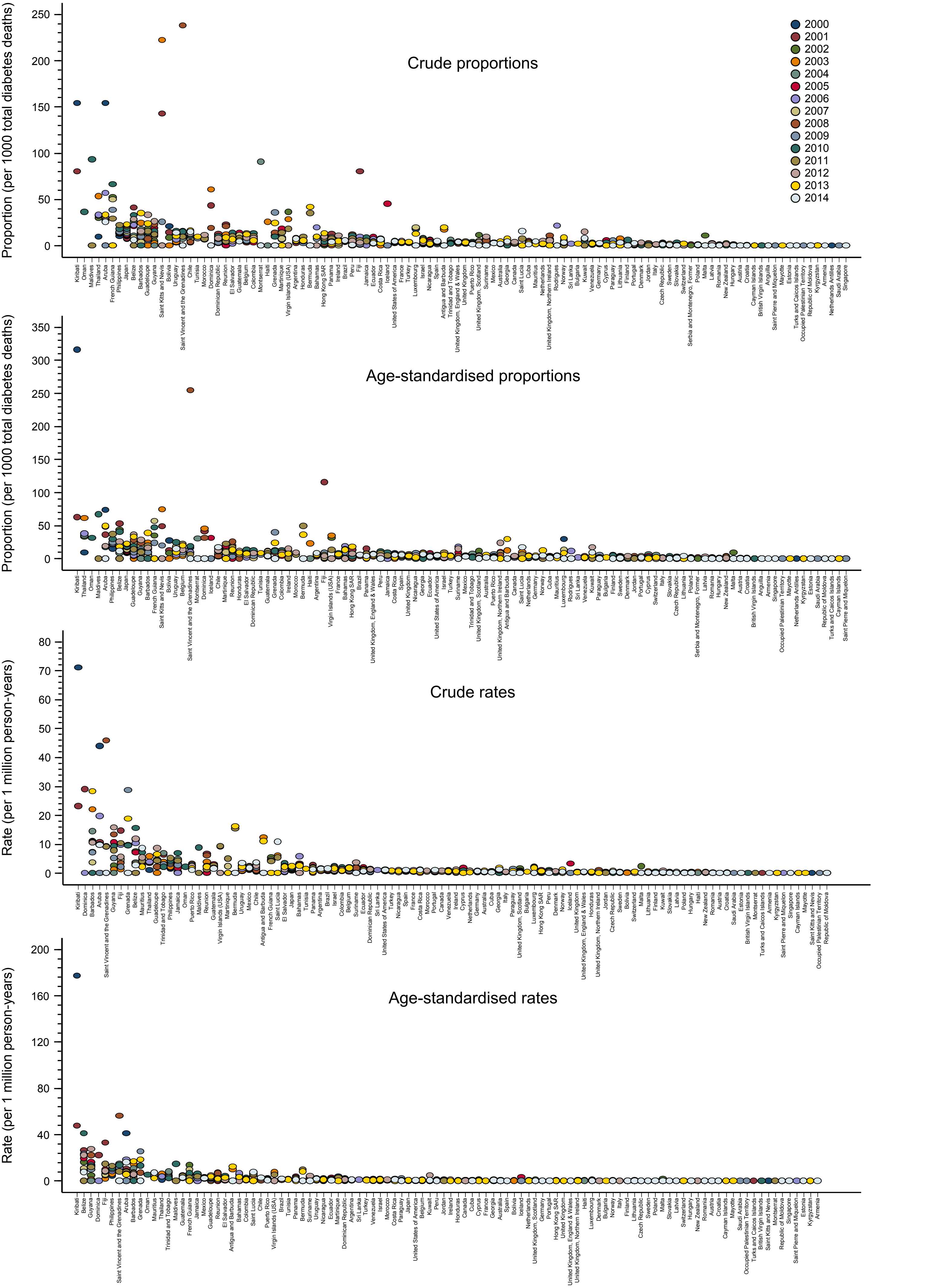

Coutries are sorted by decreasing age-standardised proportions and rates (ESM Figure 3).

ESM Figure 5: Age–standardised odds and rate ratios

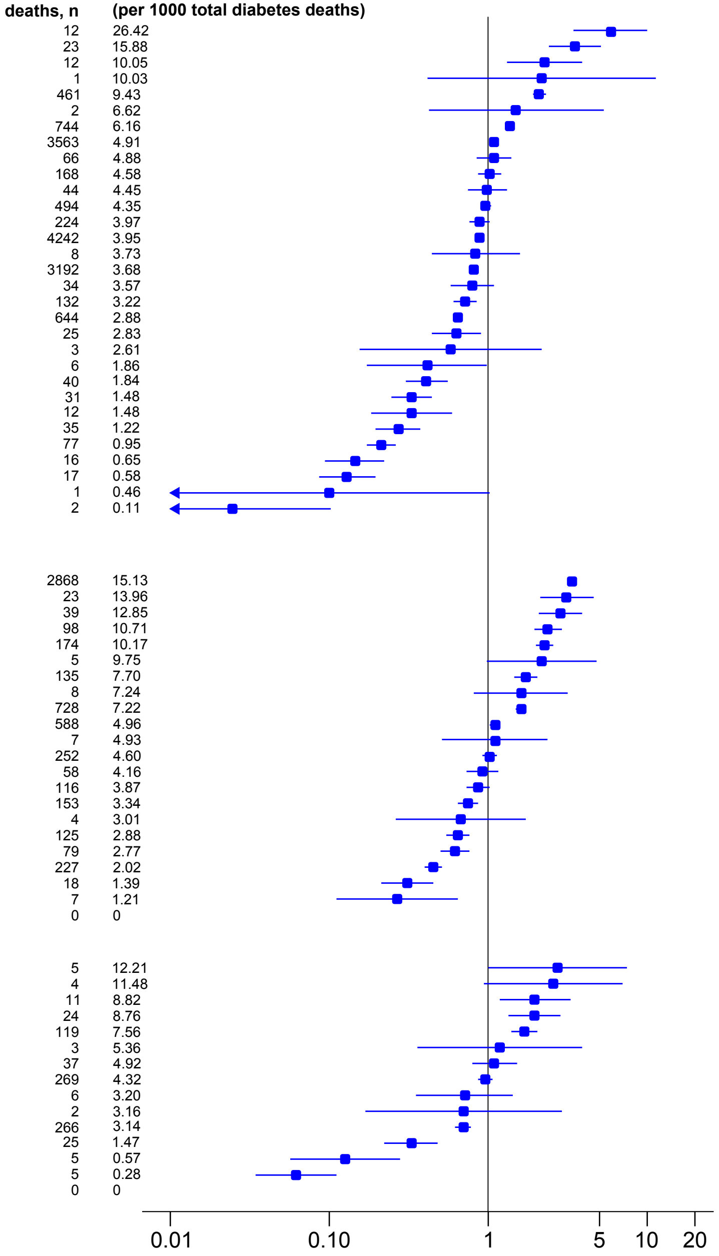

Hypoglycaemia deaths, n	Proportion (per 1000 total diabetes deaths)	Odds ratio (95% CI)	Country
			**All years available**
12	26.42	5.88 (3.45, 10.02)	Aruba
23	15.88	3.53 (2.43, 5.14)	Belize
12	10.05	2.24 (1.30, 3.86)	Saint Vincent and the Grenadines
1	10.03	2.16 (0.41, 11.34)	Montserrat
461	9.43	2.10 (1.92, 2.30)	Chile
2	6.62	1.50 (0.42, 5.33)	Bermuda
744	6.16	1.37 (1.28, 1.47)	Argentina
3563	4.91	1.09 (1.06, 1.13)	Brazil
66	4.88	1.09 (0.85, 1.39)	Panama
168	4.58	1.02 (0.87, 1.19)	Peru
44	4.45	0.99 (0.74, 1.32)	Costa Rica
494	4.35	0.97 (0.89, 1.05)	Spain
224	3.97	0.88 (0.77, 1.01)	Ecuador
4242	3.95	0.88 (0.85, 0.91)	United States of America
8	3.73	0.83 (0.44, 1.57)	Suriname
3192	3.68	0.82 (0.79, 0.85)	Mexico
34	3.57	0.79 (0.58, 1.09)	Scotland
132	3.22	0.72 (0.60, 0.85)	Puerto Rico
644	2.88	0.64 (0.60, 0.68)	Germany
25	2.83	0.63 (0.44, 0.90)	Norway
3	2.61	0.58 (0.15, 2.16)	Luxembourg
6	1.86	0.41 (0.17, 0.97)	Kuwait
40	1.84	0.41 (0.30, 0.55)	Paraguay
31	1.48	0.33 (0.24, 0.44)	Sweden
12	1.48	0.33 (0.18, 0.59)	Finland
35	1.22	0.27 (0.20, 0.37)	Czech Republic
77	0.95	0.21 (0.17, 0.26)	Poland
16	0.65	0.14 (0.09, 0.22)	Romania
17	0.58	0.13 (0.09, 0.19)	Hungary
1	0.46	0.10 (0.01, 1.03)	Malta
2	0.11	0.02 (0.01, 0.10)	Croatia
			**One year unavailable**
2868	15.13	3.37 (3.24, 3.50)	Japan
23	13.96	3.11 (2.11, 4.58)	Guadeloupe
39	12.85	2.86 (2.09, 3.93)	Barbados
98	10.71	2.38 (1.97, 2.88)	Uruguay
174	10.17	2.26 (1.99, 2.57)	Belgium
5	9.75	2.17 (0.98, 4.84)	Dominica
135	7.70	1.71 (1.45, 2.03)	El Salvador
8	7.24	1.61 (0.82, 3.16)	Grenada
728	7.22	1.61 (1.49, 1.73)	Colombia
588	4.96	1.10 (1.03, 1.19)	France
7	4.93	1.10 (0.52, 2.35)	Bahamas
252	4.60	1.02 (0.92, 1.14)	England and Wales
58	4.16	0.93 (0.74, 1.17)	Nicaragua
116	3.87	0.86 (0.73, 1.02)	Israel
153	3.34	0.74 (0.64, 0.86)	Australia
4	3.01	0.67 (0.26, 1.71)	Saint Lucia
125	2.88	0.64 (0.54, 0.76)	Netherlands
79	2.77	0.62 (0.50, 0.77)	Cuba
227	2.02	0.45 (0.39, 0.51)	Venezuela
18	1.39	0.31 (0.21, 0.45)	Switzerland
7	1.21	0.27 (0.11, 0.64)	Lithuania
0	0	-	Kyrgyzstan
			**Two years unavailable**
5	12.21	2.72 (0.99, 7.43)	French Guiana
4	11.48	2.57 (0.94, 7.02)	Saint Kitts and Nevis
11	8.82	1.97 (1.17, 3.29)	Martinique
24	8.76	1.95 (1.34, 2.85)	Reunion
119	7.56	1.68 (1.40, 2.02)	Dominican Republic
3	5.36	1.18 (0.36, 3.89)	Virgin Islands (USA)
37	4.92	1.09 (0.79, 1.52)	Hong Kong SAR
269	4.32	0.96 (0.87, 1.07)	United Kingdom
6	3.20	0.71 (0.35, 1.42)	Northern Ireland
2	3.16	0.70 (0.17, 2.91)	Antigua and Barbuda
266	3.14	0.70 (0.62, 0.78)	Canada
25	1.47	0.33 (0.22, 0.48)	Denmark
5	0.57	0.13 (0.06, 0.28)	New Zealand
5	0.28	0.06 (0.03, 0.11)	Austria
0	0	-	Anguilla

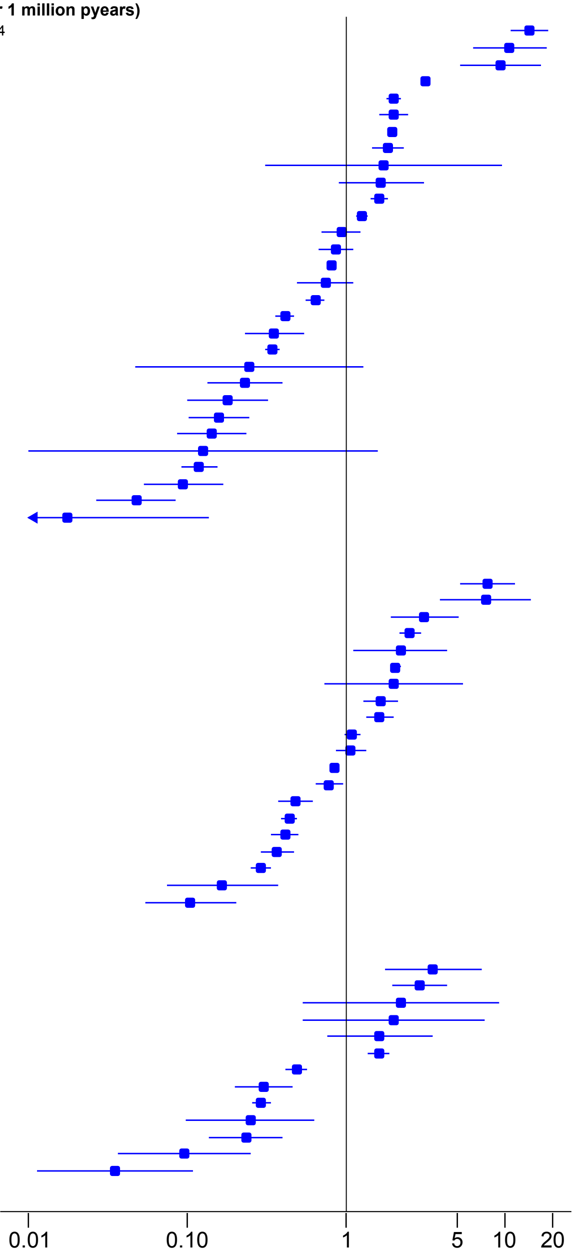

Hypoglycaemia deaths, n	Rate (per 1 million pyears)	Incidence rate ratio (95% CI)	Country
			**All years available**
23	11.34	14.45 (10.97, 19.03)	Belize
12	8.51	10.84 (6.41, 18.34)	Saint Vincent and the Grenadines
12	7.43	9.48 (5.26, 17.07)	Aruba
3192	2.48	3.16 (3.05, 3.26)	Mexico
461	1.58	2.01 (1.82, 2.22)	Chile
132	1.57	2.00 (1.62, 2.47)	Puerto Rico
3563	1.54	1.96 (1.90, 2.03)	Brazil
66	1.45	1.85 (1.48, 2.32)	Panama
2	1.38	1.74 (0.31, 9.73)	Bermuda
8	1.31	1.68 (0.90, 3.12)	Suriname
224	1.28	1.64 (1.45, 1.84)	Ecuador
744	1.00	1.27 (1.17, 1.38)	Argentina
44	0.74	0.94 (0.71, 1.25)	Costa Rica
40	0.68	0.87 (0.68, 1.12)	Paraguay
4242	0.64	0.82 (0.79, 0.85)	United States of America
6	0.59	0.75 (0.50, 1.12)	Kuwait
168	0.51	0.65 (0.57, 0.74)	Peru
494	0.33	0.42 (0.36, 0.47)	Spain
34	0.28	0.36 (0.23, 0.54)	Scotland
644	0.27	0.35 (0.31, 0.39)	Germany
3	0.19	0.25 (0.05, 1.29)	Luxembourg
25	0.18	0.23 (0.13, 0.40)	Norway
12	0.14	0.18 (0.10, 0.32)	Finland
35	0.13	0.16 (0.10, 0.25)	Czech Republic
31	0.11	0.14 (0.09, 0.24)	Sweden
1	0.09	0.13 (0.01, 1.59)	Malta
77	0.09	0.12 (0.09, 0.16)	Poland
17	0.07	0.10 (0.05, 0.17)	Hungary
16	0.04	0.05 (0.03, 0.08)	Romania
2	0.01	0.02 (0.00, 0.14)	Croatia
			**One year unavailable**
39	6.19	7.88 (5.28, 11.77)	Barbados
8	6.00	7.62 (3.92, 14.81)	Grenada
23	2.48	3.15 (1.92, 5.17)	Guadeloupe
135	2.00	2.55 (2.19, 2.96)	El Salvador
7	1.75	2.23 (1.13, 4.41)	Bahamas
728	1.63	2.07 (1.95, 2.21)	Colombia
4	1.60	2.03 (0.74, 5.54)	Saint Lucia
98	1.30	1.65 (1.28, 2.12)	Uruguay
58	1.29	1.64 (1.35, 2.00)	Nicaragua
227	0.87	1.10 (0.99, 1.23)	Venezuela
116	0.85	1.08 (0.87, 1.35)	Israel
2868	0.67	0.86 (0.81, 0.91)	Japan
174	0.62	0.79 (0.64, 0.97)	Belgium
79	0.38	0.49 (0.38, 0.63)	Cuba
588	0.35	0.44 (0.40, 0.50)	France
153	0.33	0.42 (0.34, 0.51)	Australia
125	0.29	0.37 (0.29, 0.47)	Netherlands
252	0.23	0.29 (0.25, 0.34)	England and Wales
7	0.13	0.17 (0.07, 0.37)	Lithuania
18	0.08	0.11 (0.05, 0.20)	Switzerland
0	0	-	Kyrgyzstan
			**Two years unavailable**
5	2.81	3.56 (1.77, 7.19)	French Guiana
24	2.29	2.92 (1.96, 4.36)	Reunion
2	1.76	2.22 (0.53, 9.19)	Antigua and Barbuda
3	1.55	2.01 (0.54, 7.52)	Virgin Islands (USA)
11	1.28	1.64 (0.76, 3.52)	Martinique
119	1.27	1.62 (1.38, 1.90)	Dominican Republic
266	0.39	0.49 (0.42, 0.57)	Canada
37	0.24	0.31 (0.20, 0.47)	Hong Kong SAR
269	0.23	0.30 (0.26, 0.34)	United Kingdom
6	0.20	0.25 (0.10, 0.63)	Northern Ireland
25	0.18	0.24 (0.14, 0.40)	Denmark
5	0.07	0.10 (0.04, 0.25)	New Zealand
5	0.03	0.04 (0.01, 0.11)	Austria

Standardised odds and rate ratios are compared to their respective overall estimates obtained combining all countries (reference, 1). Proportions and rates of hypoglycaemia-related deaths are sorted by available years and estimates; data for other countries are reported in ESM Table 4 for proportions (odds ratio) and Table 6 for rates (incidence rate ratio).

ESM Figure 6: Age-specific proportions and rates, by year

A

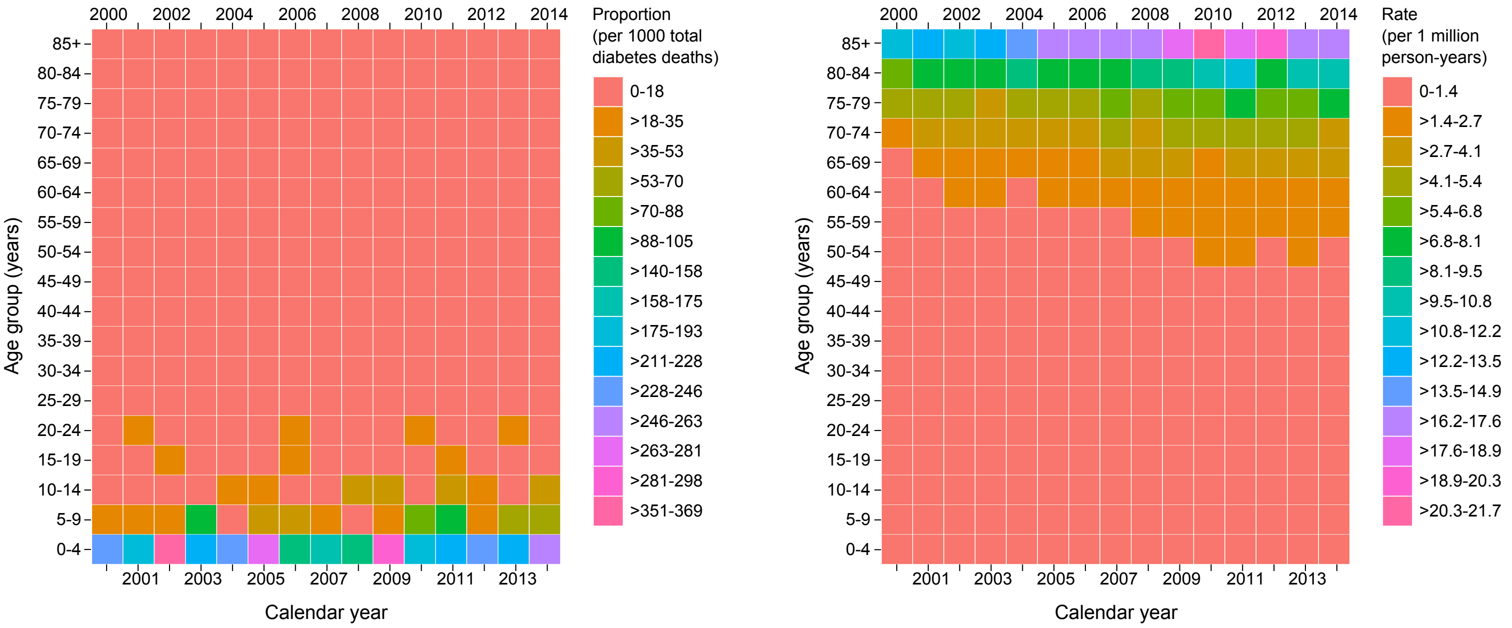

Heatmaps of age- and calendar-year specific proportions and rates (reported in discrete groups) of hypoglycaemia-related deaths.
Estimates obtained from countries with all years available.

B

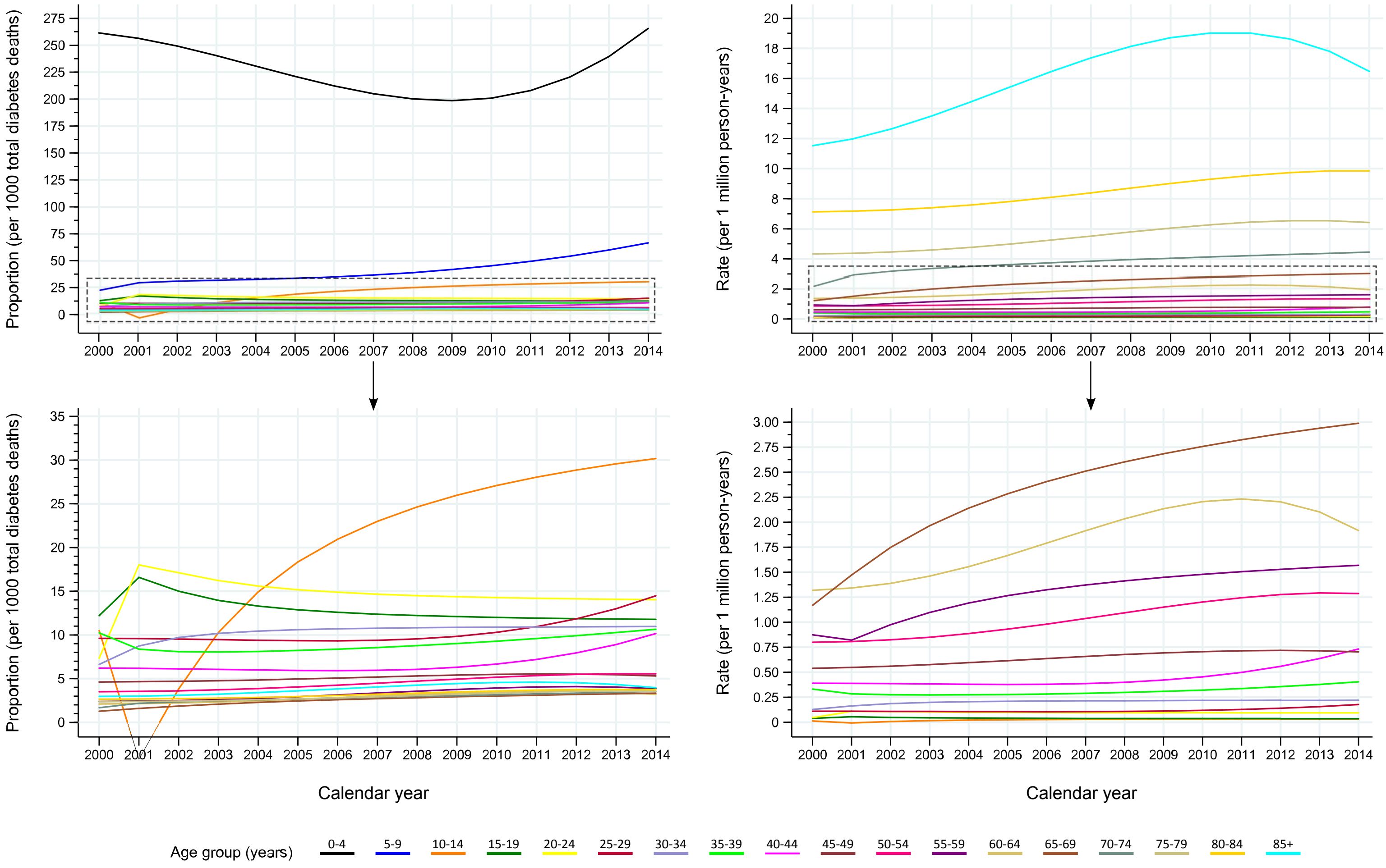

Trends of age-specific rates and proportions (reported as continuous values). Trends of rates and proportions are smoothed using fractional polynomials (values <0 for proportions in the age-group 10-14 years are related to zero events in this group in 2001, 2002, 2003). Trends within dotted areas are magnified.
Estimates obtained from countries with all years available.

ESM Figure 7: Trends of crude and age-standardised proportions for countries with data missing for one or two years

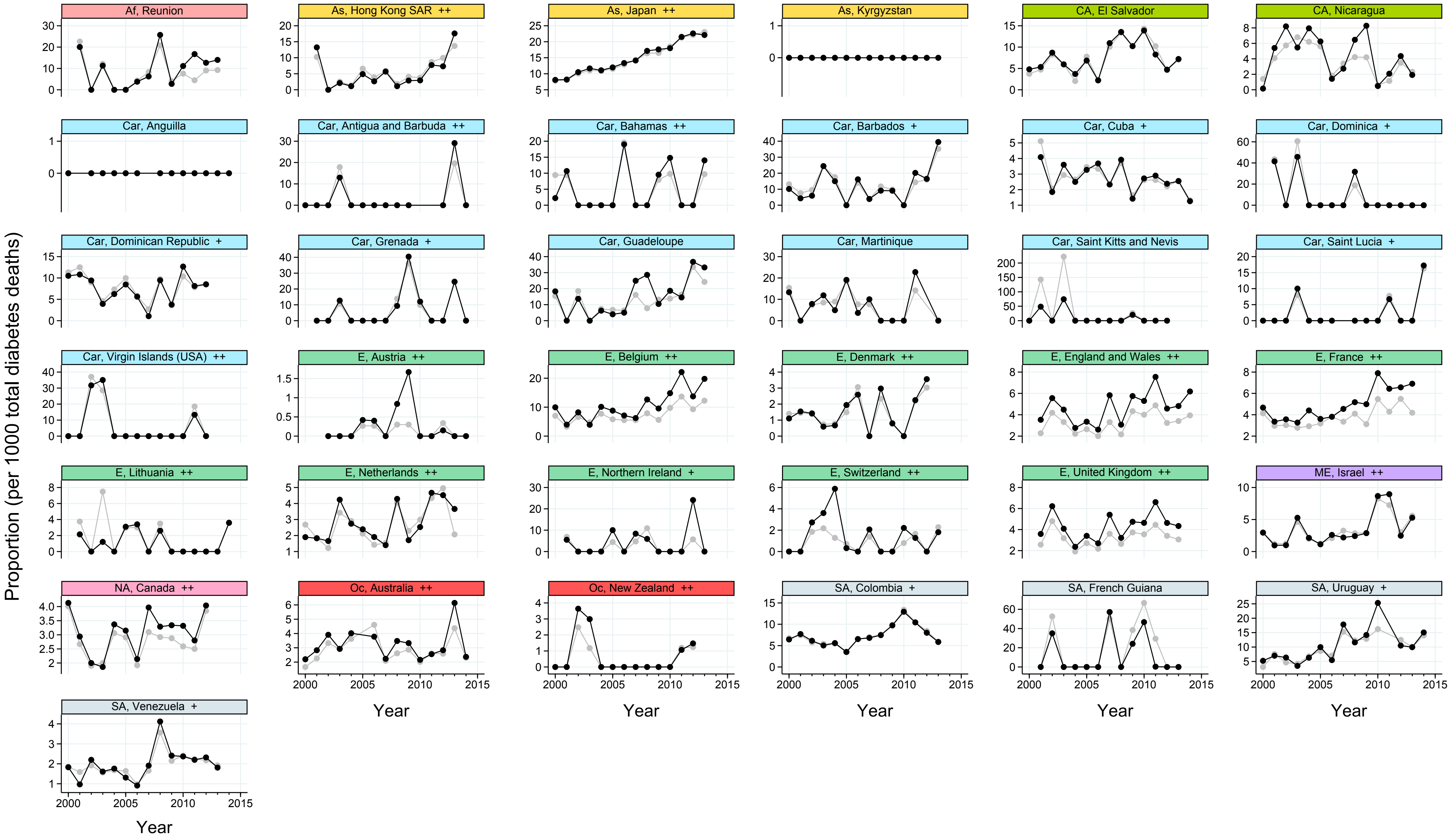

Countries are sorted by geographical region (Af, Africa; As, Asia; CA: Central America; Car, Caribbean; E, Europe; ME, Middle-East; Na, North America; Oc, Oceania; SA, South America); Socio-demographic index: +, High-middle; ++ High; no sign, not available. Grey and black circles indicate crude and age-standardised estimates of hypoglycaemia-related deaths, respectively; note the different range for y–axes.

ESM Figure 8: Trends of crude and age-standardised rates for countries with data missing for one or two years

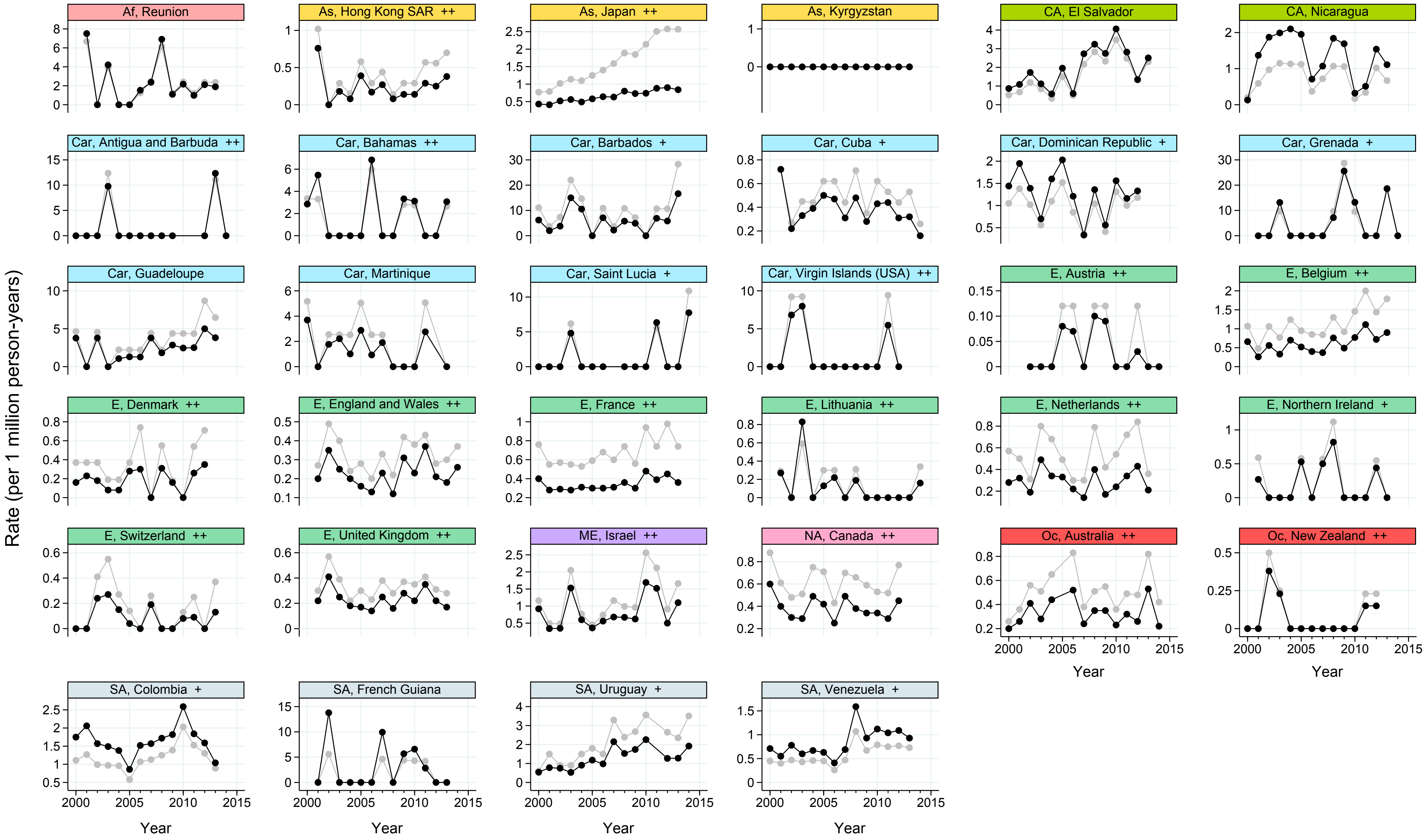

Countries are sorted by geographical region (Af, Africa; As, Asia; CA: Central America; Car, Caribbean; E, Europe; ME, Middle-East; Na, North America; Oc, Oceania; SA, South America); Socio-demographic index: +, High-middle; ++ High; no sign, not available. Grey and black circles indicate crude and age-standardised estimates of hypoglycaemia-related deaths, respectively; note the different range for y–axes.
